# Genus* Miliusa*: A Review of Phytochemistry and Pharmacology

**DOI:** 10.1155/2019/8314693

**Published:** 2019-08-14

**Authors:** Ninh The Son

**Affiliations:** Institute of Natural Products Chemistry, Vietnam Academy of Science and Technology (VAST), 18 Hoang Quoc Viet, Cau Giay, Hanoi, Vietnam

## Abstract

**Background:**

Genus* Miliusa* (family Annonaceae), widely distributed in mainland Asia and Australia to New Guinea, has been employed in both traditional herbal uses and pharmacological medicines. Original research articles related to this genus are now available, but supportive reviews highlighting phytochemical and pharmacological aspects are now insufficient.

**Objective:**

This account is an overview of most of the compounds isolated from this genus, along with their pharmacological evaluations.

**Conclusion:**

A vast amount of data showed that genus* Miliusa* contained various classes of secondary metabolites. Herein, more than two hundred constituents were isolated, comprising alkaloids, geranylated homogentisic acids, flavonoids, lignans, neolignans, terpenoids, acetogenins, styryls, lactones, phenolics, amides, alcohols, and furfural derivatives. Novel miliusanes and bicyclic lactones have been remarkable characteristics of* Miliusa* plants. Essential oils from these plants were also detected, with a high amount of *β*-caryophyllene. Numerous* in vitro* biological researches on, for example, anticancer, antifungal, antimycobacterial, anti-inflammation, and cardiac activity, especially in terms of cytotoxicity, using either isolated compounds or plant extracts, implied that* Miliusa* phytochemical components now set out to have a key role in pharmacological development.* M. smithiae* ethyl acetate extract and its flavonoid ayanin (**75**) inhibited the growth of MCF-7 cell line comparable with positive control ellipticine. (+)-Miliusol (**72**) stimulated* in vivo* anticancer experiment against HCT116 xenograft mouse tumor following the p21-dependent induction of cellular senescence mechanism.

## 1. Introduction 

People around the world have been extensively using herbal plants and their products for healthcare objectives. As can be seen, the aromatic medicinal plants have been extensively researched as an important resource of commercial drugs because of their wide traditional uses and pharmacological potencies [1]. Natural products are also recognized to be among the richest resources for new drugs and/or drug leaders due to their high structural diversity as they are not available throughout synthetic pathways [2]. Genus* Miliusa* (family: Annonaceae) comprises about 60 species and is widely native throughout India and Bhutan to Australia and New Guinea, but mostly found in many Asia countries such as Vietnam, Thailand, and China [3].

More than thirty newly rare secondary metabolites belong to derivatives of geranylated homogentisic acid; in particular, the serial novel miliusanes I-XXXI could be seen as characteristic signals to recognize plants from genus* Miliusa*.

Phytoconstituents derived from* Miliusa* plants were subjected to cytotoxic activity, acetylcholinesterase inhibition, activation of cardiac myosin ATPase, anticancer, antifungal, antibacterial, antimalaria, anti-inflammation, anti-herpes, and antioxidant activity [4–14].

In Southeast Asian traditional medicines,* M. balansae* species was used for gastropathy and glomerulonephropathy,* M. velutina* was recommended as tonic and aphrodisiac medicine, or with Thai people,* M. thorelii* species, also known as “Maa-Dam”, was applied to analgesic treatment [7, 8].

Secondary metabolites from medicinal plants of genus* Miliusa* are renowned for traditional uses and pharmaceutical potentials. However, there have not been specific reviews to assess the value of this genus, to the best of our knowledge. The current paper deals with most researches over the past 20 years related to* Miliusa *species and has given a great insight into the botanical description, the correlated chemical isolated compounds in phytochemical aspect, and their role in pharmacological applications. Databases used to search for literature mostly rely on the Plant List, SCI-Finder, Google Scholar, the Web of Science, Scopus, Hindawi, Bentham Science, Science Direct, PubMed, Chemical Abstracts, ACS journals, Springer, Taylor Francis, Wiley Online Library, Thieme Medical Publishers, and IOP Science.

## 2. Botanical Description

(i)* Nomenclature*: According to a database of the Plant List (www.Theplantlist.org, 2019), the following acceptable names of thirteen* Miliusa* species were listed at a level of high confidence:* M. balansae* Finet & Gagnep.,* M. bannaensis* X.L. Hou,* M. brahei* (F. Muell.) Jessup,* M. glochidioides* Hand.-Mazz.,* M. horsfieldii* (Bennett) Baill. ex Pierre,* M. indica* Lesch. ex A.DC.,* M. macropoda* Miq.,* M. prolifica* (Chun & F.C. How) P.T. Li,* M. sclerocarpa* (A.DC.) Kurz,* M. sinensis* Finet & Gagnep.,* M. tenuistipitata* W.T. Wang,* M. traceyi* Jessup, and* M. velutina* (A.DC.) Hook.f. & Thomson [15].

Besides known nineteen Thai species, from morphological point of view, seven new species were found in Thailand:* M. fragrans* Chaowasku & Kessler sp. nov.,* M. hirsuta* Chaowasku & Kessler sp. nov.,* M. intermedia* Chaowasku & Kessler sp. nov.,* M. nakhonsiana* Chaowasku & Kessler sp. nov.,* M. sessilis* Chaowasku & Kessler sp. nov.,* M. thailandica* Chaowasku & Kessler sp. Nov, and* M. umpangensis* Chaowasku & Kessler sp. nov. [16]. By using DNA-barcoding analysis and the morphological comparisons with the two species* M. pumila *Chaowasku and* M. filipes* Ridl.,* M. chantaburiana *Damthongdee & Chaowasku was recorded as a new species, growing in Bangkok, Thailand [17]. In the same way, an allied member of* M. indica* Leschen, named* M. jainii *Goel at Sharma, sp. nov., was discovered in South Andaman, India, and three new species,* M. cambodgensis* sp. nov.,* M. astiana*, and* M. ninhbinhensis* spp. nov., were reported to grow in Cambodia and Vietnam, respectively [3, 18].

(ii)* Phylogeny:* Genus* Miliusa* belongs to the tribe Miliuseae of the subfamily Malmeoideae of the pantropical family Annonaceae [16, 19]. Plants of this genus established a close relationship with two genera,* Hyalostemma* Wall and* Saccopetalum* Benn., and a distinguishing feature between them can be the different number of ovules (*Hyalostemma* only one,* Miliusa* two, and* Saccopetalum* more than two) [19].

(iii)* General morphology*: The plants exist in shape of shrub or tree (up to* ca*. 40 m high). The wood appeared yellow when fresh but became darker on exposure; the parenchyma was in fine tangential lines, forming a network with the narrow to moderately broad to broad rays. The mature leaf in* M. horsfieldii*, for instance, indicated elliptic to ovate shape [19].* Miliusa *species have also been shown to be associated with the following characteristics: equally sized sepals and outer petals both of which are much smaller than the inner petals, a densely hairy torus, miliusoid stamens, i.e. stamens without conspicuously dilated connective tissue covering the thecae, and four part-lamellate ruminations of the endosperm [16].

(iv)* Distribution*: Genus* Miliusa*, until now, has been reported to consist of about 60 species [17], distributed throughout India and Bhutan to Australia and New Guinea, but mostly found in mainland Asia [19].

## 3. Phytochemical Investigation

**Figure 1:**
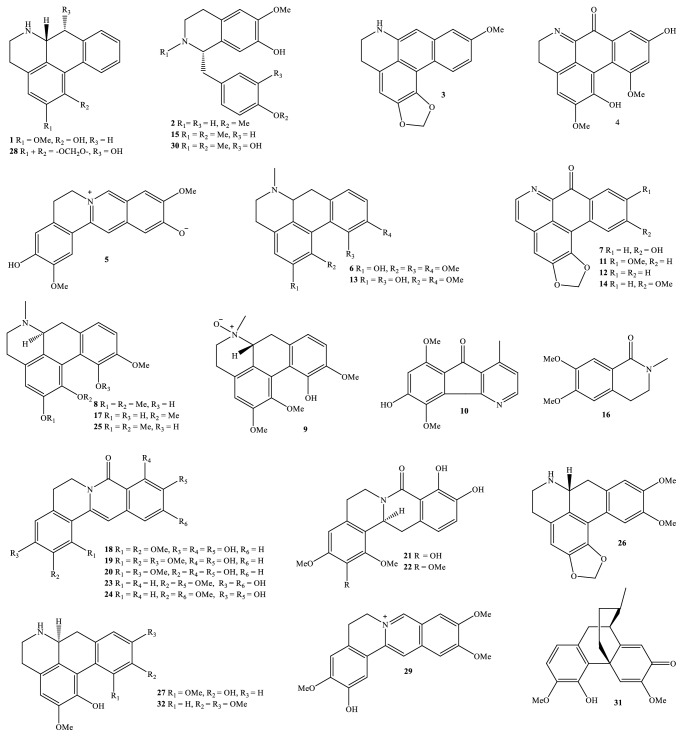
Alkaloids from* Miliusa* species.

**Figure 2:**
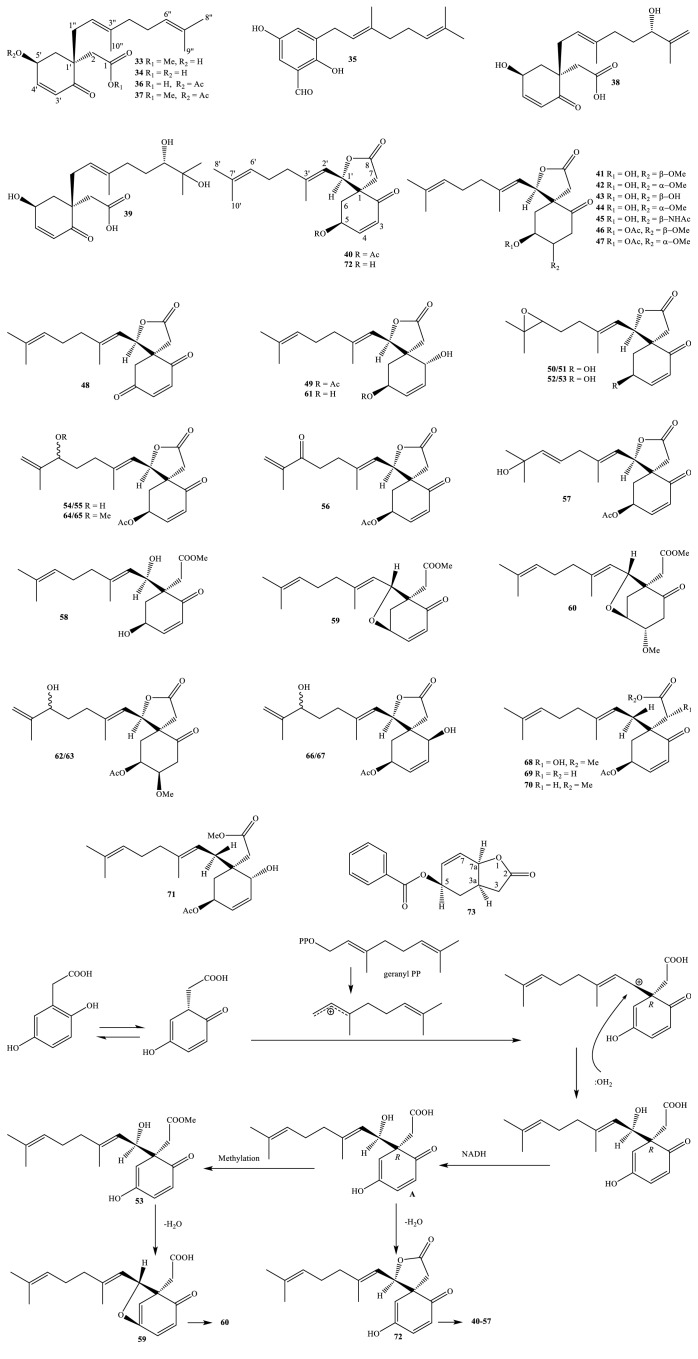
Geranylated homogentisic acid derivatives from* Miliusa* species and their plausible biogenetic pathway.

**Figure 3:**
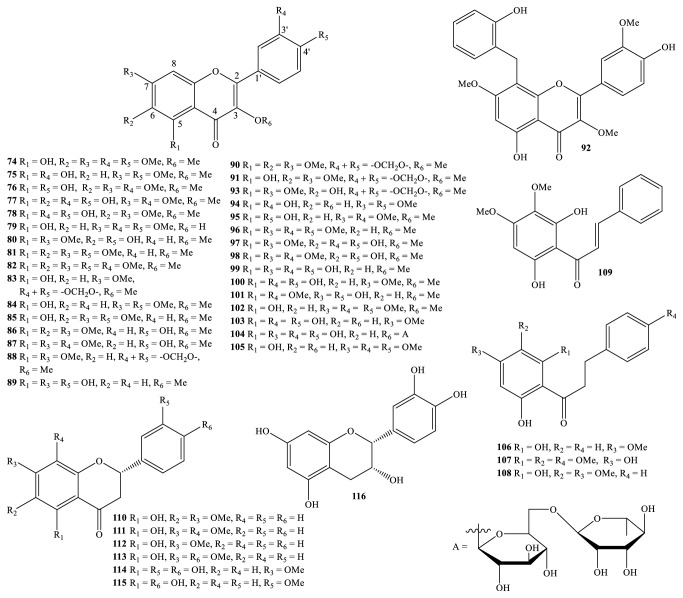
Flavonoids from* Miliusa* species.

**Figure 4:**
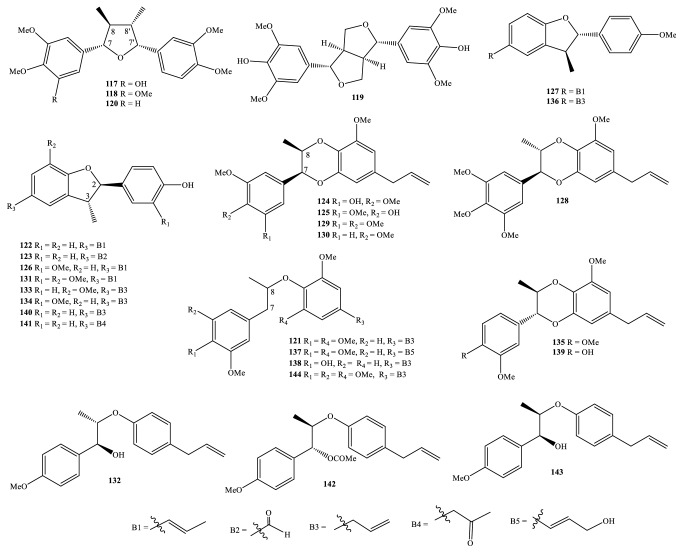
Lignans and neolignans from* Miliusa* species.

**Figure 5:**
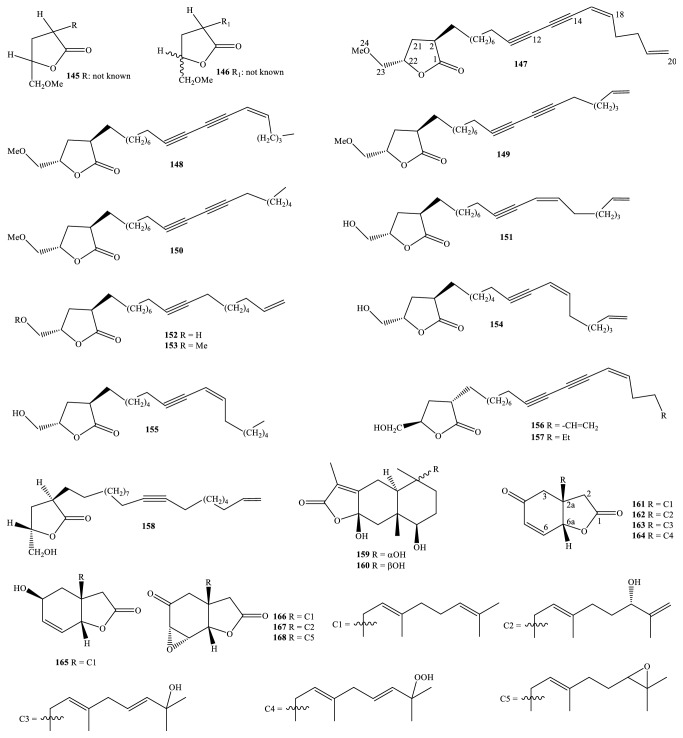
Acetogenins and lactones from* Miliusa* species.

**Figure 6:**
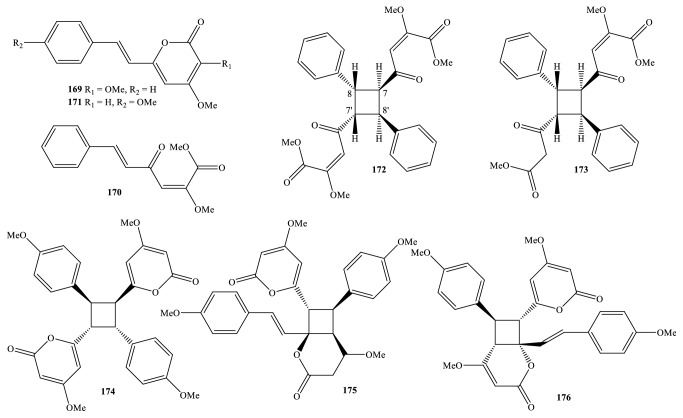
Styryls from* Miliusa* species.

**Figure 7:**
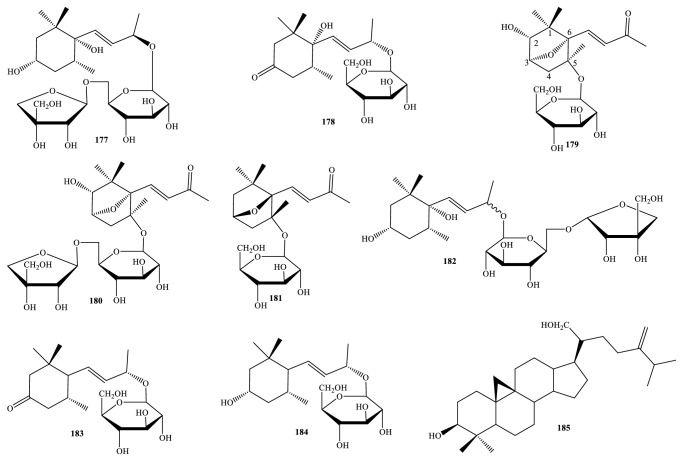
Terpenoids from* Miliusa* species.

**Figure 8:**
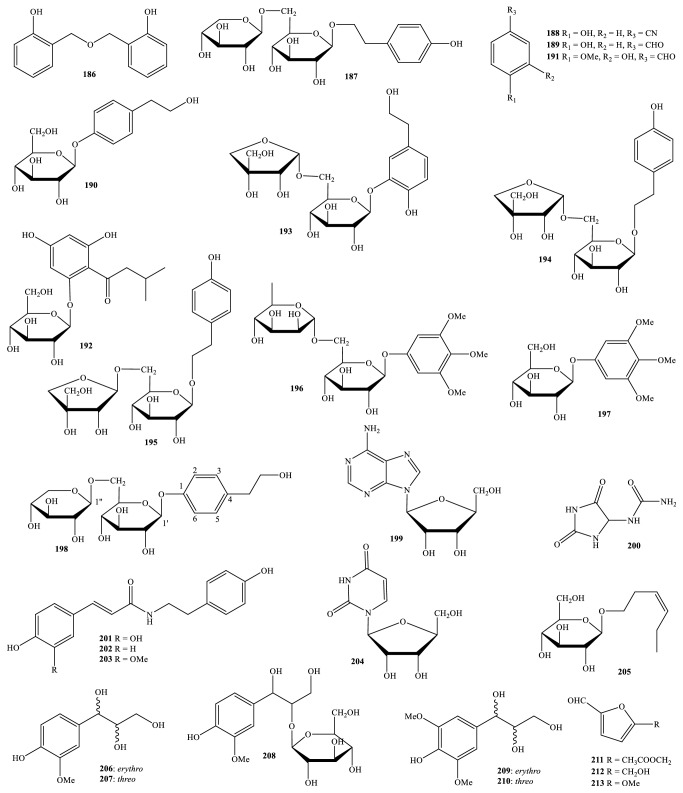
Mono-phenols, amines, amides, alcohols, and furans from* Miliusa* species.

**Figure 9:**
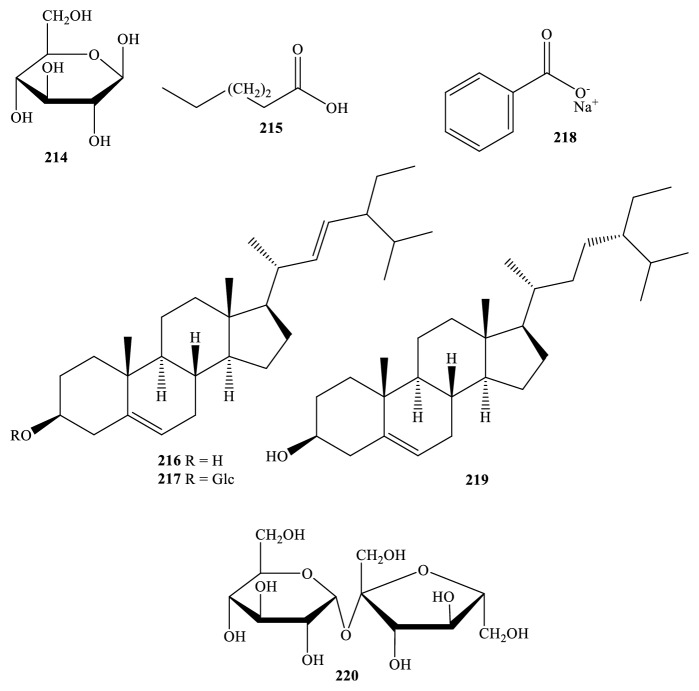
Other types from* Miliusa *species.

Nowadays, the methods and the processes of the isolation and elucidation of naturally occurring compounds from the medicinal plants have received heavy supports from the modern techniques, such as high performance liquid chromatography (HPLC), gas chromatography-mass spectrum (GC-MS), nuclear magnetic resonance (NMR), ultraviolet-visible (UV-Vis), infrared (IR), optical rotation (OR), and circular dichroism (CD) spectroscopies [1, 20]. We, herein, set out an updated phytochemical account of all isolated metabolites from* Miliusa* species, principally based on chromatographic procedures. Many works have been carried out on the phytochemical investigations of several parts of ten plants, namely,* M. balansae*,* M. *CF.* banacea*,* M. cuneata*,* M. fragrans*,* M. mollis*,* M. sinensis*,* M. smithiae*,* M. thorelii*,* M. umpangensis*, and* M. velutina* [4, 7, 21–26]. Two hundred twenty secondary metabolites were recorded and presented in Table 1 and Figures 1–9. The names of the isolated compounds have been prepared following the arrangement of alphabetical words. In addition, Table 2 indicated a list of main essential oils from five studied species:* M. baillonii*,* M. brahei*,* M. horsfieldii*,* M. traceyi*, and* M. sinensis* [27–29]. Isolated metabolites of* Miliusa* species were classified into a wide range, including alkaloids, geranylated homogentisic acids, flavonoids, lignans and neolignans, acetogenins, styryls, mono-phenols, terpenoids, amines and amides, alcohols, furans, and other types. The first group of thirty-two compounds** 1-32** was referred to as alkaloids [4, 7, 21–26]. Forty-one metabolites from compound** 33** to compound** 73** could be conveniently classified into the group of homogentisic acid derivatives [7, 13, 14, 30–34]. The structures** 74-116** were recognized to be flavonoids [7, 8, 11, 25, 30, 33, 35–38]. Lignans and neolignans were also found in the plants of genus* Miliusa* and were actually described by the state of next compounds** 117-144** [7, 9, 10, 24]. Due to the remarkable features, the serial compounds** 145-158** were assignable to the group of acetogenin derivatives [5, 32, 38–40]. Ten compounds** 159-168** can be seen as lactones [11, 38], and eight constituents** 169-176** belonged to styryl derivatives [31, 32, 38, 41]. Terpenoids included the structures** 177-185** [11, 25, 37, 42]. The reports of mono-phenols and their glycosides from* Miliusa* species are now available elsewhere, but, herein, they were summed up in a total of thirteen compounds** 186-198** [11, 24, 30, 32, 42]. Six amines and amides** 199-204** [7, 8, 26], six alcohols** 205-210** [11], three aldehydes type furfurals** 211-213** [32], and last compounds** 214-220** [25, 26, 32, 41] have been identified as the remaining metabolites present in genus* Miliusa*.

### 3.1. Alkaloids

With natural product substances, alkaloidal compounds were famous long ago. The proportion of alkaloids has been found to deeply depend on the typical parts of the plants and environmental effects. For example, Aniszewski (2007) suggested that a large percentage of alkaloids reached up to 10-25% from the higher plants [1].* Miliusa* species also provide a rich alkaloidal source. Up to now, over thirty alkaloidal constituents** 1-32** were recorded, involved in the previous phytochemical investigations on several plants,* M. balansae*,* M. *CF.* banacea*,* M. mollis*,* M. sinensis*,* M. thorelii*, and* M. velutina*, but mostly found in* M. cuneata *(Figure 1) [4, 7, 21–26, 37]. Among them, eleven constituents, namely, compounds** 4-5**,** 7**,** 9**, and** 18-24**, were new in nature. Liriodenine (**12**) was likely to be familiar with plants from genus* Miliusa* and widely distributed in* M. balansae* stem,* M. cuneata *stem and leaf,* M. mollis *twig,* M. sinensis* leaf and branch, and* M. velutina *stem bark [21, 23–26, 37]. With the exception of new compounds and two known others,** 12** and** 28**, it was remarked that the remaining compounds were isolated from this genus for the first time.* Miliusa* alkaloids can be found in formulating a variety of main skeletons, such as aporphine and oxo-aporphine backbones in respective asimilobine (**1**) and 10-hydroxyliriodenine (**7**), tetrahydroisoquinoline and quinolone in respective coclaurine (**2**) and N-methylcorydaldine (**16**), azafluorenone in kinabaline (**10**), dihydroprotoberberine and oxo-protoberberine in respective 2,10-dimethoxy-3,11-dihydroxy-5,6-dihydroprotoberberine (**5**) and miliusacunine A (**18**), benzylisoquinoline in reticuline (**30**), or morphinan in salutarine (**31**).

Taking the newly isolated alkaloids into consideration, two new oxo-aporphine and dihydroprotoberberine alkaloids** 4-5**, in addition to thirteen known others (Table 1), were obtained from 95% ethanol extract of air-dried and powdered stem and leaf of* M. cuneata* [21]. One of the immensely value aspects of 2,10-dimethoxy-3,11-dihydroxy-5,6-dihydroprotoberberine (**5**) is that this compound possessed the positive and negative charges in nitrogen and oxygen atoms, respectively [21]. Khan and Kumar (2015) suggested that alkaloids with bipolar charges had a tendency to bind to the serum proteins better than that of neutral compounds [43].

Following the outcomes in the isolation and NMR-structural elucidation, 10-methoxyliriodenine (**14**) was a known alkaloid, but its 10-hydroxylated derivative** 7** was determined to be a new oxo-aporphine alkaloid in nature; both of these two compounds were precipitated out of the MeCOEt extract (2.8 g) of* M*. CF.* banacea* species [4]. Likewise, isocorydine (**8**), especially its new unusual derivative (+)-isocorydine *α*-*N*-oxide (**9**), has successfully been separated from extracts of* M. velutina* stem bark [22, 23].

An additional significance in phytochemical works related to plants is that the leaf of* M. cuneata* species is likely to be a rich source of oxo-protoberberine alkaloids. In 2005, five new alkaloids of type oxo-protoberberine were isolated from the acetone extract of* M. cuneata *leaf, trivially named miliusacunines A-E (**18-22**) [7].

Finally, three alkaloids, consisting of two new dihydro-oxo-protoberberine derivatives miliusathorines A-B (**23-24**) and known one (−)-norushinsunine (**28**), have been purified from the combined extract between stem and root of* M. thorelii* species [8].

### 3.2. Geranylated Homogentisic Acid Derivatives

Phenolic acids of type homogentisic acids are usually detected in both terrestrial plants and bacterial pathogenic strains [44, 45]. Homogentisic acids indicated the significant antioxidant and anti-inflammatory capacities, but the excess accumulation of these can cause “alkapton” symptom in the human body [45, 46].

Considerable attention should be paid to the novel class of geranylated homogentisic acid derivatives from plants of genus* Miliusa*. Among the total forty-one isolated compounds** 33-73**, secondary metabolites** 40-73** were novel and relatively rare compounds in nature while a number of isolates** 35-39** were new in literature.

General features were highlighted in the chemical structures** 33-34** and** 36-72**; that is, carbonylation and geranylation often occurred at carbons C-2 (or C-2′) and C-1 (or C-1′), respectively (Figure 2). Furthermore, double bonds might be located at carbons C-3 and C-4 (or C-3′ and C-4′); hydroxylation, methoxylation, or acetoxylation was normally observed at carbon C-5 (or C-5′).

Of isolated compounds** 40-72**, most of these unique structures might possibly be formed by a rare C-18 skeleton, containing a characteristic *γ*-lactone spiro-ring system. Five-member *γ*-lactone ring were geranylated in the structures** 40-57**,** 61-67**, and** 72**, but were found to be opened in the structures** 58-60** and** 68-71**. Two isolated compounds** 59-60** also contained a tetrahydrofuran ring. Additionally, the combination of NOE effect observations, Mosher reactions, and X-ray measurements allowed for determining the absolute configuration, in which 1*R*,5*S*,1′*R*-form and 5*β*-orientation were suitable for the group of compounds** 40-72** [14]. ^13^C-NMR provided the evidence of chemical shifts *δ*_C_, thereby showing that, at carbons C-1, C-5, and C-1′ of compounds containing spiro-ring system, *δ*_C_ reached* ca*. 52.0-56.0 ppm,* ca*. 63.0-68.0 ppm, and* ca*. 26 ppm, respectively.

As part of an interdependent work, more recently, Promgool et al. (2019) reported that chromatographic separation of extracts of* M. velutina *fruit and flower has resulted in isolating and elucidating five new rare geranylated homogentisic acid derivatives, miliusanal (**35**) and miliusanones A-D (**36-39**), in addition to known ones, methyl 2-(1′*β*-geranyl-5′*β*-hydroxy-2′-oxocyclohex-3′-enyl) acetate (**33**) and 2-(1′*β*-geranyl-5′*β*-hydroxy-2′-oxocyclohex-3′-enyl) acetic acid (**34**) [32]. New compound** 35** indicated the property of a phenolic aldehyde with CHO (*δ*_C_ 196.1 ppm in solvent CDCl_3_), whereas new compounds** 38-39** were significantly made of 6′′-hydroxylated groups (*δ*_C_ 75.6-77.4 ppm in solvent CDCl_3_ + CD_3_OD).

Novel (+)-miliusate (**40**) was one of the interesting constituents of Vietnamese plant* M. balansae* [31]. After that, it was reported appearing in two other species:* M. sinensis *leaf, twig and flower, and* M. umpangensis* leaf [14, 19, 33, 34]. So far, the methanol-water extract (95:5, v/v) of Vietnamese plant* M. balansae* leaf and branch has shown to comprise one novel compound, (+)-miliusol (**72**), and one new natural product, miliusolide (**73**) [30]. In NOE interactions H-3a to H-5 and H-7a were key evidence to determine these three protons with oriented* cis-*shape and the absolute configuration was established as 3a*S*,5*S*,7a*R *in compound** 73**.

Novel geranylated homogentisic acids derived* Miliusa* plants were more commonly referred to by their trivial name. By using Table 1 and Figure 2, we continue to make comments relating to a serial number of (+)-miliusanes I-XXXI (**41-71**). Secondary metabolites** 41-60** were separated from dichloromethane extract of the other Vietnamese plant* M. sinensis *leaf, twig, and flower, whereas the remaining members** 61-71**, once again, derived from* M. balansae* species [13, 14]. As shown in Figure 2, the plausible biogenetic pathway explained why these compounds were classified as geranylated homogentisic acids, in which the first step involved the combination between precursor homogentisic acid and geranyl unit of geranyl diphosphate (geranyl PP). Obviously, the methylation of intermediate product** A** produced compound** 53** while lactone ring-cyclization and dehydrate applied to** A** would give** 72**; compound** 53** was then joined to epoxide ring-cyclization reaction and dehydrated to form** 59** [14].

Taken together, serial compounds** 40-72** were useful biomarkers for either genus* Miliusa* or family Annonaceae, and they also accounted for the close relationship between the two species* M. balansae *and* M. sinensis*.

### 3.3. Flavonoids

Now we do take a point of crucial information in mentioning another class of* Miliusa* metabolites. Phytochemical investigations on* Miliusa* species also proved the existence of flavonoids. Flavonoids were detected in leaf, twig, branch, stem, or root of nine plants:* M. balansae*,* M. cuneata*,* M. fragrans*,* M. mollis*,* M. sinensis*,* M. smithiae*,* M. thorelii*,* M. umpangensis*, and* M. velutina*, to date (Table 2). Herein, we draw a list of forty-three isolated compounds** 74-116** from* Miliusa* species. Chemical index also exhibited that* Miliusa *flavonoids can be divided into several main groups: flavonols** 74-105**, chalcones** 106-109**, flavanones** 110-115**, and flavan** 116** (Table 1 and Figure 3) [7, 8, 11, 25, 30, 33, 35–37]. More than thirty flavonols were found but flavones and isoflavones were absent, to the best of our knowledge. Likewise, flavanones and isoflavanones have not been recorded yet.

The most important information to be gained from structural features is that isolated flavonoids derived from* Miliusa* species were generated as* mono*-flavonoid derivatives. The phenomenon of methoxylation occurred at carbon C-3 of most isolated flavonols. Normally, flavonols and flavanones were associated with substituents at carbons C-5, C-6, C-7, C-8, C-3', and C-4' by hydroxy and methoxyl groups.

Despite the fact that the known flavonoids** 74**,** 77-82**,** 84-91**,** 96-99**,** 101-103**,** 105-107**,** 109**, and** 114-115** are abundant in the plant kingdom, these compounds were reported from* Miliusa* species for the first time. Rutin (**104**) was recognized to be only flavonol glycoside isolated from plants of genus* Miliusa*. In the meantime, (−)-epicatechin (**116**) was also a unique flavan to be found.

*M. thorelii* species seemed to be a rich supply of flavonols. Bioguided assay and fractionation of extracts of leaf, stem, and root afforded one new natural product, miliusathorone (**92**), in addition to nineteen known ones,** 74**,** 80-83**,** 85-86**,** 88-91**, and** 95-102** [8]. New metabolite** 92** had the characteristic remark with chemical shifts of -O-CH_2_-O- [*δ*_H_ 6.06, *δ*_C_ 101.8]. Interestingly, new metabolite named miliufavol (**92**) was derived from methanol-water (95:5, v/v) extract of air-dried ground* M. balansae* leaf and branch [35], in which this compound was an uncommon flavonol by combining of pachypodol (**95**) and benzyl unit at carbon C-8.

Four chalcone derivatives, 2′,6′-dihydroxy-4′-methoxydihydrochalcone (**106**), 4′,6′-dihydroxy-2′,3′,4-trimethoxydihydrochalcone (**107**), dihydropashanone (**108**), and pashanone (**109**), were significant constituents of the two Vietnamese plants* M. balansae *and* M. sinensis* [25, 31, 37]. In contrast to** 109**, three compounds** 106-108** belong to dihydrochalcone, and compound** 107** was a new isolated compound in literature. Isolated flavanones** 110-113** were also considered as main components of Vietnamese* M. balansae *and* M. sinensis*, but two compounds, final derivatives 7-*O*-methyleriodictyol (**114**) and sakuranetin (**115**), were only detected in* M. velutina *leaf up to now [38].

### 3.4. Lignans and Neolignans

We continuously provide the next phytochemical profiles of the other class of isolated compounds. Starting with the deepest aim to find biologically active molecules from genus* Miliusa*, four lignans** 117-120** and twenty-four neolignans** 121-144** have also been isolated (Table 1 and Figure 4). In addition, these phytochemicals from genus* Miliusa* originated from three parts, leaf, stem, and twig, of two main species,* M. fragrans* and* M. mollis*, but occasionally found in* M. cuneata *species [8–10, 24].

It is possible to set up a clear arrangement for backbones of either lignans or neolignans.* Miliusa* lignans were able to form up to two main skeletons, tetrahydrofuran lignan (compounds** 117-118** and** 120**) and 7,9′:7′,9-diepoxylignan (compound** 119**). In the case of neolignans, three main scaffolds can be found, namely, 8.*O*.4′-neolignan (compounds** 121**,** 132**,** 137-138**, and** 142-144**), 7.*O*.3′,8.*O*.4′-neolignan (**124-125**,** 128-130**,** 135**, and** 139**), and dihydrobenzofuran skeleton (compounds** 122-123**,** 126-127**,** 131**,** 133-134**,** 136**, and** 140-141**). Two skeletons of 8.*O*.4′-neolignan and 7.*O*.3′,8.*O*.4′-neolignan were only represented in the two Thai species* M. fragrans* and* M. mollis*, thereby suggesting the close relationship between them [9, 10, 24].

Besides geranylated homogentisic acids, oxo-protoberberine alkaloids, and flavonols, chemical constituents of* M. cuneata *leaf were also in association with the presence of the well-known lignan (+)-syringaresinol (**119**) [7]. In 2013, sixteen secondary metabolites, being isolated from methanol extracts of Thai* M. fragrans *leaf and stem in the work of Sawasdee and partners, were described as three lignans** 117-118**,** 120** and thirteen neolignans** 121**,** 124-125**,** 128-131**,** 135**,** 137-139**, and** 143-144** [9]. The new lignan (+)-3-hydroxyveraguensin (**117**) and its relatives** 118** and** 120** have been structurally established as 7*S*,8*S*,7′*R*,8′*S*-configuration (*cis*-H-7/H-7′,* cis*-H-7′/H-8′,* trans*-H-7/H-8, and* trans*-H-8/H-8′). The new neolignans** 124-125**,** 128**,** 135**, and** 139** and two known ones** 129-130** shared the same structure of three parts, 5-methoxyl-phenylpropanoid unit, 2-methyl-1,4-dioxane unit, and phenyl ring linkage to carbon C-7. However, the absolute configurations were elucidated as 7*S*,8*R* for** 124-125** and** 129-130**, 7*S*,8*S *for** 128**, and 7*R*,8*R *for** 135** and** 139**, but the stereochemistry for new 8.*O*.4′-neolignans** 137-138** and their analogs** 138** and** 144** could not be determined.

In the two years 2010 and 2013, Sawadee and partners provided the results of the phytochemical isolation and NMR-structural elucidation of Thai* M. mollis*, in which one new neolignan of type dihydrobenzofuran (2*S*,3*S*)-2,3-dihydro-2-(4-methoxyphenyl)-3-methyl-5-[1(*E*)-propenyl]benzofuran (**127**), one new 8.*O*.4′-neolignan (7*S*,8*S*)-*threo*-Δ^8′^-4-methoxyneolignan (**132**), and two known others** 123** and** 126** were isolated from twig; five new neolignans of type dihydrobenzofuran 7-methoxymiliumollin (**133)**, 3′-methoxymiliumollin (**134**), 4′-*O*-methylmiliumollin (**136**), miliumollin (**140**), miliumollinone (**141**); one new 8.*O*.4′-neolignan miliusanollin (**142**); and one known other (**123**) were derived from leaf [9, 10].

Neolignans of type dihydrobenzofuran from Thai plants have been shown to be associated with 2*R*,3*R*-absolute configuration (*trans*-H-2/H-3) in groups** 122-123**,** 126**,** 133-134**, and** 140-141** and 2*S*,3*S*-absolute configuration (*cis*-H-2/H-3) in** 127** and** 136**, while new compound** 132** set up 7*S*,8*S*-model when compared to 7*R*,8*R*-model in** 142** and 7*S*,8*R*-model in** 143**.

### 3.5. Acetogenins and Lactones

A bit of the attractive phytochemical outcome arose from the class of isolated acetogenins. As we know, acetogenins and their analogs are now remarkable characteristics of the family Annonaceae [47]. From Table 1 and Figure 5, isolated acetogenins existed in bark, stem bark, flower, and leaf of* M. velutina* species and they were new compounds except for goniothalamusin (**158**). The most striking feature is that these isolated compounds were able to form up to one or two triple bonds in a long aliphatic side chain terminated by *γ*-hydroxy (or *γ*-methoxyl)-*γ*-lactone unit, methyl group, or double bond.

Earlier phytochemical report by Jumana and coauthors (2000) showed that acetogenins A-B (**145-146**) obtained from Bangladeshis* M. velutina* species reached up to 0.00154% and 0.008% of extract weight, respectively [5]. However, NMR data of long alkyl side chain of these two compounds remain unknown. Paying attention to the result of phytochemical study on* n*-hexane extract of Thai* M. velutina *stem bark, based on repeating chromatographic columns on silica gel and sequential LiChrosorb RP-18 column techniques, we found that eight new compounds, alphabetically named cananginones A-I (**147-155**), have been successfully isolated as colorless viscous liquids [40]. Taking cananginone A (**147**) as an example, this new C23 linear olefinic acetogenin is accompanied by a variety of significant chemical shifts [(*γ*-methoxyl)-*γ*-lactone] unit established at *δ*_C_ 179.6 (C-1), *δ*_C_ 39.2 (C-2), *δ*_C_ 30.4 (C-21), *δ*_C_ 76.7 (C-22), *δ*_C_ 74.3 (C-23), and *δ*_C_ 59.5 (C-24); two double bonds and two triple bonds occurring at *δ*_C_ 65.1-146.6 ppm] [40]. Additionally, the positive OR value of +17.4 (*c* 0.206, CHCl_3_) evidently confirmed 2*R*,22*S*-configuration compared with that of goniothalamusin (**158**) [+14.6 (*c* 0.206, CHCl_3_)] [40].

Miliusolide and its dihydro derivatives** 156-157** were also new derivatives of acetogenin detected in* M. velutina* stem bark [39]. Unfortunately, the trivial name ‘miliusolide' was used for both metabolites** 73** and** 156** [30, 39].

In the light of phytochemical research, Wongsa and partners (2017) continuously provided the outcome relative to Thai* M. velutina* species (Figure 5) [38]. From* n*-hexane and ethyl acetate extracts of leaf of this plant, a rare class of eight bicyclic lactones with a C18 carbon backbone, trivially named velutinones A-H (**161-168**), were isolated. Similar to acetogenins, these compounds were collected with the physical property of colorless viscous liquids. Furthermore, they had shown the same feature in chemical structures, by which geranyl groups are located at carbon C-2a and the relative configuration at carbons C-2a and C-6a was* syn*-model (2a*R*,6a*R*). In general, it is worth concluding that olefinic acetogenins with terminated *γ*-lactone and 2-geranylated bicyclic lactones indicated a great crucial role in chemotaxonomic aspect to recognize* M. velutina*. Lactones in genus* Miliusa *were also found in* M. balansae*; the two known compounds curcolide (**159**) and serralactone (**160**) were two components of the leaf of this plant, collected from Vietnam [11].

### 3.6. Styryls

Most of secondary metabolites of interest to chemists pointed out that styryl derivatives were found in genus* Miliusa*. Styryls presented as the significant constituents of the two species* M. balansae* and* M. velutina* [31, 32, 38, 41]. As shown in Table 1 and Figure 6, the three* mono*-styryls** 169-171** and the five* bis*-styryls** 172-176** have been updated. It is worth noting that yangonin (**171**) was, for the first time, reported from genus* Miliusa*, while the seven remaining isolates** 169** and** 172-176** were reported to be new compounds in nature.

The shrub tree* M. balansae *is widely distributed in Vietnam and China; chromatographic examination of the polar extract of leaf and branch of this plant yielded the two new mono-styryls 3,4-dimethoxy-6-styryl-pyran-2-one (**169**) and (2*E*,5*E*)-2-methoxy-4-oxo-6-phenyl-hexa-2,5-dienoic acid methyl ester (**170**) [31].

Regarding isolated compounds of type* bis*-styryls, the general chemical structure was designated by cyclobutyl nucleus, while side chains were made up of phenyl rings, *α*-pyrone rings, and *α*,*β*-unsaturated ketones.

In order to identify bioactive constituents, phytochemical investigation has been carried out on methanol-water extract (95:5, v/v) of Vietnamese* M. balansae* leaf and branch, which continuously demonstrated the existence of two bulk new* bis*-styryls miliubisstyryls A-B (**172-173**). Although NMR data of** 172** were not completely assigned, the key NOE evidence proposed that the relative configurations of** 172** and** 173** were identical, being* trans*-form for H-7/H-8 and H-7′/H-8′, together with* cis*-form for H-8/H-7′ and H-7/H-8′.

As mentioned above,* M. velutina *is a good reservoir of unique bicyclic lactones. From this plant, the three new* bis*-styryls velutinindimers A-C (**174-176**) were also separated [38]. According to this article, OR value approximately reached zero ([*α*]_D_ +0.08 (*c* 0.63, MeOH-CHCl_3_ 3:1)] and no Cotton effect was observed in CD spectrum which can be responsible for the symmetrical property of velutinindimer A (**174**) (compound containing a symmetrical plane). Similarly, the combination of the assignments of ^1^H, ^13^C-NMR spectroscopic signals and the correlations in 2D-NMR data, as well as the most utilization of advantageous techniques such as the CD and X-ray measurements that considered velutinindimers B-C (**175-176**), were two racemic compounds and the relative configurations of these compounds were 5′*S*,6′*R*,7*S*,8*S* in compound** 175** ([*α*]_D_ +0.08 (*c* 0.63, MeOH-CHCl_3_ 5:1)) and 5′*R*,6′*S*,7*R*,8*S* in compound** 176** ([*α*]_D_ +0.03 (*c* 0.23, MeOH-CHCl_3_ 9:1)).

### 3.7. Terpenoids and Phenols

*M. balansae* species seems to be the most crucial objective in the contents of phytochemical researches related to plants of* Miliusa* species. Following the application of the variously chromatographic methods, norsesquiterpenoids of type megastigmanes,* mono*-phenols, and their glycosides have been determined as characteristics of genus* Miliusa*, especially* M. balansae* species. Eight terpenoids** 177-185** and thirteen phenolic compounds** 186-198** were summarized in Table 1 and Figure 7, which were newly isolated compounds or isolated for the first time from genus* Miliusa* [11, 25, 30, 32, 37, 42].

Herein, mono-saccharide units of type *β*-D-glucopyranosyl parts and disaccharides units of type *α*-D-apiofuranosyl-(1→6)-*O*-*β*-D-glucopyranosyl, *β*-D-apiofuranosyl-(1→6)-*O*-*β*-D-glucopyranosyl, *β*-xylopyranosyl-(1→6)-*O*-*β*-D-glucopyranoside, *α*-L-rhamnosyl-(1→6)-*β*-D-glucopyranosyl moieties are glycone parts linked to aglycones of terpenoids and phenols, whereas aglycones of phenols are mostly structurally formed by phenylethanoid nucleus.

Together with the one known terpenoid alangionoside B (**177**) and the four known phenolic glycosides cuchiloside (**187**), osmanthuside H (**195**), 1-(*α*-L-rhamnosyl-(1→6)-*β*-D-glucopyranosyloxy)-3,4,5-trimethoxybenzene (**196**), and 3,4,5-trimethoxyphenol-*β*-D-glucopyranoside (**197**), the phytochemical investigation of Chinese* M. balansae* species afforded one new megastigmane glycoside miliusoside C (**182**) and two new* mono*-phenols of type phenylethanoid glycosides miliusosides A-B (**193-194**) from 80% ethanol extract of dried stem [42]. The newly isolated compound** 182** differed from its similar structure** 177** in the orientation of D-apiosyl unit (*α*-form in** 182** and *β*-form in** 177**).

In Vietnamese* M. sinensis *leaf and branch, 24-methylencycloartane-3*β*,21-diol (**185**) was the only triterpenoid reported to date, whereas the small-simple molecules 4-hydroxybenzonitrile (**188**), 4-hydroxybenzaldehyde (**189**), and isovanillin (**191**) were* mono*-phenols from* M. velutina *fruit [25, 32, 37].

Phytochemical analysis of methanol extract of Vietnamese* M. balansae* leaf has permitted the isolation and determination of the three new megastigmane glycosides milbasides A-C (**179-181**), in addition to the three analogs ampelopsisionoside (**178**), myrsinionosides A and D (**183-184**), and one glucosylated phenol 1-(3-methylbutyryl)phloroglucinol-glucopyranoside (**192**) [11]. Structures** 179-181** shared the same* E*-geometrical shape of double bond outside; in addition, due to the negative Cotton effect at around 240 nm (Δ*ε* ranged from –1.73 to –3.27), the absolute configurations of** 179-180** were 2*R*,3*S*,5*S*,6*S*, while compound** 181** was proposed as 3*S*,5*R*,6*R *(Δ*ε*_245nm_ +3.27). In the same manner, chemical shift of methylene group of* bis*(2-hydroxyphenyl) methyl ether (**186**) was higher than that of hydroxymethyl alcohol by 8 ppm, implying that compound** 186** was a new symmetrical ether [30].

This review updated a phytochemical result of* M. mollis*; the twig of this plant also contained the two phenolic glycosides icariside D2 (**190**) and tyrosol 1-*O*-*β*-xylopyranosyl-(1→6)-*O*-*β*-D-glucopyranoside (**198**) [24]. The highlight in phenyl ring of aglycone of new compound** 198** corresponded to A_2_B_2_ spin system [*δ*_H_ 7.10 (2H, d,* J* = 8.6 Hz, H-3 and H-5) and *δ*_H_ 6.95 (2H, d,* J* = 8.6 Hz, H-2 and H-6)], with glycone being remarked with two anomeric protons at *δ*_H_ 4.73 (1H, d,* J* = 7.3 Hz, H-1′) and *δ*_H_ 4.17 (1H, d,* J *= 7.6 Hz, H-1′′).

### 3.8. Amine, Amide, Alcohol Derivatives and Miscellaneous Types

A phytochemical survey conducted by Yu and partners (2009) pointed out that* M. balansae* leaf, collected from China, has also been composed of one amine adenine riboside (**199**), and two amide allantoin (**200**), and uridine (**204**) (Figure 8) [26]. Although these compounds are now available in the natural plants, they were isolated as single compounds from genus* Miliusa* for the first time. Utilizing silica gel (63-200 *μ*m) and sephadex LH-20 columns in the chromatographic isolation, three tyramine derivatives,* N-trans*-caffeoyltyramine (**201**),* N-trans*-coumaroyltyramine (**202**), and* N-trans*-feruloyltyramine (**203**), have been purified from acetone of* M*.* cuneata* air-dried twig [7]. Since then, acetone extract of* M. thorelii* stem and root was demonstrated to contain two isolates,** 201** and** 203** too [8].

Vietnamese* M. balansae* species might have been considered as a rich resource of diverse compounds. Based on the evidence of phytochemical findings and NMR explanations, six well-known alcohol derivatives,** 205-210**, were further obtained from the leaf of this plant for the first time [11]. Among them, *β*-D-glucopyranoside (*Z*)-3-hexenol (**205**) was a glycosylation of long* n*-alkyl side chain alcohol, while the five remaining ones** 206-210** were categorized as two pairs of* erythro* and* threo* isomers of glycerols and one other glucosylated glycerol. They were* erythro*-guaiacylglycerol (**206**),* threo*-guaiacylglycerol (**207**),* erythro*-1-*C*-syringylglycerol (**209**),* threo*-1-*C*-syringylglycerol (**210**), and (L)-guaiacyl glycerol 2′-*O*-*β*-D-glucopyranoside (**208**), respectively. There were also records of these compounds from genus* Miliusa* for the first time. As can be seen, the difference of chemical components from Vietnamese and Chinese M.* balansae* species has been depending on geographic factor more often.

Besides the presence of the rare compounds of type homogentisic acid derivatives, the ethyl acetate extract of air-dried* M. velutina* fruit also consisted of furfural derivatives, in which three small molecules, 5-acetyloxymethylfurfural (**211**), 5-hydroxymethylfurfural (**212**), and 5-methoxyfurfural (**213**), were isolated [32]. Despite the fact that these compounds were formulated with a simple pattern, they were recorded from the plants of genus* Miliusa* for the first time.

As shown in Figure 9 and Table 1, the common chemical compounds of type phytosterols can be found in several* Miliusa* plants. For instance,* M. balansae *stem was reported to contain *β*-sitosterol (**216**), and its glucoside** (217**), or stigmasterol (**219**); this one turned out to be one of the components from both* M. sinensis *leaf and branch and* M. velutina* fruit and flower [25, 32].

Chromatographic separation of the extracts from* M. balansae *stem also led to the isolation and determination of two mono-saccharide D-glucose (**214**) and sucrose (**220**) whereas* M. balansae *leaf and branch were accompanied by the existence of fatty acid octacosanoic acid (**215**) and benzoate sodium (**218**) [26, 32, 41].

### 3.9. Essential Oils

For a long time, there have been researches related to essential oils, which have frequently played an important role and been regarded as a branch of either phytochemistry or pharmacological products findings [1]. Many studies focused on the applications providing multifunction in healthcare problems, alternative medicines, food and drink manufacturers, or household cleaning products. Normally, the distillation method, often by using water steam, is a prompt and efficacious way; in addition, the extractions of solvent or florasols have always been used depending on the raw materials. The systematic GC and GC-MS techniques are usually being used to identify volatile individuals.* Miliusa* species are also among the largest sources of this specific chemical compound. In the review of all circumstances, following the results of ethno-geographic distribution, species property, part use, extraction method, and GC-MS technical analysis, the results of studying essential oils of* Miliusa* species were compiled in Table 2.

Essential oils studies on* Miliusa *plants are quite limited. There have been only three reports to date. Among forty-six members identified from essential oils of fresh leaf of, Quang Binh, Vietnamese* M. baillonii* species, the main constituent* Z*-citral reached the highest amount of 41.2% [28].* M. sinensis* species, collected from Nghe An, Vietnam, is also likely to be a rich resource of essential oils. From this plant, 67.1% of a total of 95.1% of essential oils were sesquiterpene hydrocarbons [29]. Significantly, *α*-humulene and *β*-caryophyllene could be seen as the main components of oils of these two Vietnamese* Miliusa* plants (Table 2). In the same manner, the properties of plants, environmental factors, and collection time accounted for the differential components in essential oils of three, Queensland, Australian* Miliusa *plants,* M. brahei*,* M. horsfieldii*, and* M. traceyi* [27]. As can be seen from Table 2, these three species yielded oils, in which terpenoids predominated. *α*-Humulene, *β*-caryophyllene, and bicyclogermacrene achieved more than 10% of oils of* M. brahei*, while the major sesquiterpene of type caryophyllene derivatives ranged from 12% to 20%, present in* M. horsfieldii* oils; the highest components in oils of last plant* M. traceyi* encountered were the two isomers *α*- and *β*-pinene (approximately 19%). Of particular interest, *β*-caryophyllene was found to be one of the main compounds in all the five species (Table 2), which accounted for 10% to 20% of oils of* Miliusa* species.

## 4. Pharmacological Activities

### 4.1. Cytotoxic Activity

The plants of genus* Miliusa* included sets of variously useful isolated components to the 得 experimentally cytotoxic targets. In the review of all conditions, the cytotoxic results were briefly summarized in Table 3. Earlier report in 2000 by Jumana and partners mentioned the cytotoxicity of constituents from* M. velutina* species; the LC_90_ results of tested compounds may run as acetogenin A (**145**) (LC_90_ 7.1 *μ*g/mL) > acetogenin B (**146**) (LC_90_ 14.1 *μ*g/mL) > positive control vincristine (LC_90_ 15.0 *μ*g/mL) > goniothalamusin (**158**) (LC_90_ 20.0 *μ*g/mL) [5]. Nine new acetogenins cananginones A-I (**147-155**) were found to possess the weak IC_50_ values of 16.6-129.7 *μ*M or be inactive in the cytotoxic assay against three cancer cell lines KB, MCF7, and NCI-H187, when compared to those of reference compound doxorubicin (IC_50_ 0.46-1.05 *μ*M) [40].

Huong and partners (2004b) pointed out four flavonoids,** 75-76** and** 94-95**, from Vietnamese* M. balansae* plant not just to show their powerful capacities (IC_50_ < 5.0 *μ*g/mL) in cytotoxic assay against the three cancer cell lines KB, Hep-G2, and RD, but also to emphasize that the introduction and modification of functional groups at carbon C-3, C-6, C-3′, and C-4′ were reasonable in the different results between pachypodol (**95**) and the remaining tested compounds** 75-76** and** 94** [30].

We then moved on to the demonstration of the proper agents from another Vietnamese medicinal plant,* M. sinensis*.* n*-Hexane and ethyl acetate extracts of this plant were moderately or weakly active against the four cancer cell lines MCF-7, LU, Hep-G2, and KB (IC_50_ 42.5-86.6 *μ*g/mL); in particular, their secondary metabolite liriodenine (**12**) induced the strong IC_50_ values of 2.3-2.89 *μ*g/mL towards MCF-7 and KB, but* n*-butanol extract and two isolated flavonoids, 3,5-dihydroxy-7,3′,4′-trimethoxyflavone (**79**) and 4′,6′-dihydroxy-2′,3′,4-trimethoxydihydrochalcone (**107**), did not show significant potency (IC_50_ > 128.0 *μ*g/mL) [37]. Likewise, the weak-polar extracts of type* n*-hexane and ethyl acetate extracts of Thai* M. smithiae *species generally revealed moderate cytotoxic results in the experiment with eight cell lines, P-388, KB, Col-2, MCF-7, Lu-1, T24, ASK, and Hek293, but better than the inactivation of the polar extracts of type acetone and methanol extracts [36]. It is worthy of note that ethyl acetate part and its isolated flavonoid ayanin (**75**) against MCF-7 with the IC_50_ values of 0.3-0.68 *μ*g/mL were comparable to positive control ellipticine (IC_50_ 0.37 *μ*g/mL) [36].

In a comparison between new dihydrobenzofuran neolignan 3′ methoxymiliumollin (**134**) and its analogs decurrenal (**123**), miliumollin (**140**), and miliumollinone (**141**) in the cytotoxic assay against the three cancer cell lines KB, MC7, and NCI-H187 (Table 3), methoxylation would lead to reducing the IC_50_ values but the modification of allyl group did not seem to be the way of positive signal [9]. In another case, with the same test to KB, MC7, and NCI-H187 cell lines, the cytotoxic activity of chemical constituents of* M. fragrans* induced a clear arrangement as follows: 7*S*,8*R*-8.*O*.4′-neolignan** 143** (IC_50_ 12.7-14.4 *μ*g/mL) > dihydrobenzofuran neolignan** 131** (IC_50_ 12.9-16.7 *μ*g/mL) > 7*R*,8*R*-7.*O*.3′,8.*O*.4′-neolignan** 139** (IC_50_ 16.7-23.8 *μ*g/mL) > 7*S*,8*R*-7.*O*.3′,8.*O*.4′-neolignans** 124-125** and** 130 **(IC_50_ 15.9-28.4 *μ*g/mL) > flavan** 116** (inactive) [10].

Now, it is pretty noticeable that uncommon bicyclic lactones velutinindimers A-D and F-H (**161-164** and** 166-168**) gave rise to a range of the IC_50_ values from 4.0 *μ*M to 24.1 *μ*M in inhibiting three cancer cell lines, KB, MCF-7, and NCI-H187, and Vero cell line, while three new styryl derivatives** 174-176** failed to do so [38]. As discussed above, leaf of* M. cuneata* was renowned as a reservoir of the diverse classes of secondary metabolites, but there has been a discrepancy between them in the cytotoxic assay [7]. In detail, either alkaloids, amides, flavonoids, or lignans inactivated towards two cell lines, KB and Vero, only geranylated homogentisic acid (+)-miliusol (**72**) and took part in suppressing these two cell lines with the IC_50_ values of 10.2 ± 0.1 *μ*M and 13.5 ± 0.5 *μ*M, respectively.

Phytochemical studies on* Miliusa *plants have reached certain successes with isolating and identifying the presence of geranylated homogentisic acids, but more than ever, these compounds were further set to justify the cytotoxicity.

Isolated compounds** 33-37** possessed the cytotoxic activities against the four cell lines KB, MCF-7, NCI-H187, and Vero with IC_50_ values in the range of 5.8-40.4 *μ*g/mL, and the failure of the two compounds** 38-39** (IC_50_ > 50.0 *μ*g/mL) led to a hypothetical suggestion that the modification of double bond of geranyl unit would not be considered as a good method to promote the positive signal in assay [32].

Twenty-two homogentisic acid derivatives, comprising (+)-miliusate (**40**), (+)-miliusol (**72**), and serial (+)-miliusanes I-XX (**41-60**), have been screened by the cytotoxic assay with seven cancer cell lines, KB, Lu1, Col2, LNCaP, MCF-7, HUVEC, and HL60, and the results were briefly summarized in Table 2 [14]. It is worth mentioning that in structure-biology relationship, at the dose of 20.0 *μ*g/mL, (+)-miliusane V (**45**) was reported to be a nontoxic compound (IC_50_ > 55.0 *μ*g/mL) due to acetyl amide group. When comparing between (+)-miliusate (**40**) and (+)-miliusane IX (**49**), at carbon C-2, carbonylation was better than hydroxylation, but the opposite phenomenon was observed between (+)-miliusane VIII (**48**) and (+)-miliusol (**72**) since hydroxy group transferred into carbonyl group at carbon C-5. In accordance with the above results, hydroxylation, carbonylation, and epoxidation occurred at double bond of geranyl units of (+)-miliusanes X-XVII (**50-57**); the cytotoxicity did not enhance. Last but not least, *γ*-lactone ring opening, such as in compounds (+)-miliusanes XVIII-XIX (**58-59**), was shown not to render the cytotoxicity.

As part of the ongoing effort to improve the efficacy of miliusane derivatives, recently, nineteen isolated compounds, (+)-miliusate (**40**), (+)-miliusanes I-II, IX, XIV-XV, XVII, XXI-XXI (**41-42**,** 49**,** 54-55**,** 57, 61-71**), and (+)-miliusol (**72**), were continued to submit the cytotoxic assay with three cancer cell lines HCT116, A375, and A549 [13]. The results showed the positive signals when compounds** 40-42**,** 54-55**,** 57**,** 68**, especially (+)-miliusate (**40**), and (+)-miliusol (**72**) were demonstrated to be the most active with the IC_50_ values of 1.0-5.0 *μ*M.

### 4.2. Anticancer Activity

Anticancer experiments have been so far designated as a consequence of cytotoxicity. With the GI_50_ values in the range of 0.03-4.79 *μ*M, three geranylated derivatives of homogentisic acids, (+)-miliusate (**40**), (+)-miliusane I (**41**), and (+)-miliusol (**72**), showed the potential antitumor activities towards NCI-60 panel of human cancer cell lines, but were more active with HCT116 cell line [13]. In a comprehensive analysis, the main component of* Miliusa* plants, namely, (+)-miliusol (**72**), was highly recommended to anticancer drugs development. At the end of 21st day of* in vivo* anticancer treatment, this compound (20.0 mg/kg) induced the decrease in average size of excised HCT116 xenograft mouse tumor up to 72.7%, and the mechanism may be due to p21-dependent induction of cellular senescence rather than apoptosis [13].

### 4.3. Antimalarial Activity

With regard to antimalarial activities against* Plasmodium falciparum* strains TM4 and K1, the IC_50_ values established a consistent arrangement as follows: standard compound cycloguanil (IC_50_ 0.08 ± 0.01 *μ*M and IC_50_ 31.0 ± 8.4 *μ*M) > geranylated homogentisic acid (+)-miliusol (**72**) (IC_50_ 11.1 ± 2.0 *μ*M and IC_50_ 9.1 ± 3.1 *μ*M) > new oxo-protoberberine alkaloids** 18-22**, flavones** 76** and** 87**, and amides** 201** and** 203** (IC_50_ 19.3-41.4 *μ*M and IC_50_ 10.8-54.9 *μ*M) > flavones** 83** and** 95**, lignan** 119** and amide** 202 **(inactive) [7]. It should be noted that among the four flavones** 76**,** 83**,** 87**, and** 95**, methylation at 5-OH and methoxylation at carbon C-6 can be responsible for promoting antimalaria, in contrast to the phenomenon dioxane-cyclization between two hydroxyl groups at C-3′ and C-4′, whereas hydroxylation and methoxylation at* meta*-position of caffeoyl unit induced the potential differences among the three amides** 201-203**.

In addition to antimalarial assay, two known isolated compounds** 33-34** and new one miliusanone A (**36**) inhibited the growth of* P. falciparum* with the IC_50_ values of 3.3-3.9 *μ*g/mL but better than those of analogs miliusanone A (**37**) (IC_50_ 5.2 *μ*g/mL), miliusanal (**35**), and miliusanones C-D (**38-39**) (IC_50_ > 10 *μ*g/mL) [32]. From these data, it was also concluded that among the four geranylated homogentisic acid derivatives** 33-34** and** 36-37**, the number of ester groups in the structure seemed to be the main for the outcome.

Serial new acetogenins cananginones A-I (**147-150** and** 152-155**) failed to inhibit* P. falciparum* except for cananginone E (**151**) (IC_50_ 24.4 *μ*M) [40]. Comparing between the structure** 151** and the close group of compounds** 152** and** 154-155**, dihydroxylation of double bonds and methylene reduction would not be facilitated.

Three new bulk styryl derivatives, velutinindimers A-C (**174-175**), established better IC_50_ values in the range of 5.4-6.4 *μ*M towards* P. falciparum *than new unique bicyclic lactones velutinones B-D, G, and H (**162-164** and** 167-168**) (IC_50_ 7.3-10.0 *μ*M) [38].

### 4.4. Antifungal and Antimycobacterial Activities

In the search for natural products against DNA repair mutant in the yeast strain* Saccharomyces cerevisiae*, the MeCOEt extract of* M.* CF.* banacea* root showed inhibited* rad* 52.* top 1* (IC_12_ 2000 *μ*g/mL), but failed to do so with* rad *52 and* rad*^+^ (IC_12_ > 8000 *μ*g/mL) [4]. In the meantime, new alkaloid 10-hydroxyliriodenine (**7**) and positive control camptothecin afforded the respective IC_12_ values of 72 *μ*g/mL and >20 *μ*g/mL in suppressing* rad* 52.* top 1*, being better than that of 10-methoxyliriodenine (**14**) (IC_12_ 113 *μ*g/mL).

The MIC values ranging from 4.0 *μ*g/mL to >128 *μ*g/mL were the results when using chalcone pashanone (**109**) and flavanone 5-hydroxy-6,7-dimethoxyflavanone (**110**) against thirteen human pathogenic fungi,* Candida albicans* ATCC10231,* Candida krusei *ATCC6258,* Candida lusitaniae* ATCC42720,* Candida tropicalis* ATCC13803,* Cryptococcus gattii *R265,* Cryptococcus gattii* WM276,* Cryptococcus neoformans *JEC21,* Cryptococcus neoformans* ATCC36556,* Cryptococcus neoformans *var.* grubii* H99,* Aspergillus fumigatus *ATCC16424, and* Trichophyton mentagrophytes* ATCC9533 [6].

The effect of new cananginones H-I (**154-155**) on fungal* C. albicans *has been shown to be associated with the IC_50_ values of 75.2 *μ*M and 37.4 *μ*M, respectively, but new acetogenins cananginones A-G (**147-153**) did not appear active [40]. Herein, the reason is opposite to antimalarial analysis when four structures** 151-152** and** 154-155** were considerably compared.

The global health problem in developing countries is becoming increasingly involved in growing and expanding of microbacteria. Historical records have accumulated evidence showing that the use of traditional antibiotics, which are derived from synthetic substances, is always accompanied by a long duration of treatment, high cost, and drug resistance [48]. Therefore, calls for new antibiotic drugs from natural sources in the fight against multidrug-resistant bacteria are always strategy.

In a short communication, various components from* M. tomentosa* species suppressed the growth of several kinds of bacteria and fungi, but the most significant finding is that leaf volatile oil extract reduced the growth of bacterium* Bacillus subtilis *NCIM 2250 and fungal* C. albicans* NCIM 3471 with the same MIC value of 0.62 mg/mL, being better than those of leaf aqueous extract (2.5 mg/mL and 5.0 mg/mL, respectively) [49].

Acetogenin A (**145**) and goniothalamusin (**158**) are associated with the moderate antibacterial activity with diameters of the inhibitory zone of 9-14 mm against positive Gram bacteria* Bacillus cereus*,* Staphylococcus aureus*, and* Streptococcus β-haemolyticus *and 9-11 nm against negative Gram bacteria* Salmonella typhi*,* Shigella flexneri*, and* Shigella dysenteriae*, whereas acetogenin A (**146**) only influenced* B. cereus *with diameter zone of 11 mm [5].

The MIC values ranged from 32.0 to 64.0 *μ*g/mL, which were the moderate antibacterial result of two geranylated homogentisic acids,** 33** and** 35**, repellent positive Gram bacteria* B. cereus* DMST 5040,* S. aureus* DMST 8013, and methicillin resistant* S. aureus*. Meanwhile, two other geranylated homogentisic acids,** 34 **and** 36**, and two styryls,** 171** and** 175**, were only found to be associated with the MIC values of 64.0-128.0 *μ*g/mL against* B. cereus* DMST 5040 [32]. It was, therefore, concluded that geranylated homogentisic acids seemed to be better candidates for this problem rather than styryl derivatives.

At the same time, compound** 33** revealed the MIC value of 50 *μ*g/mL to treat* Mycobacterium tuberculosis* H37Ra compared with that of the similar structures** 34-39** (MIC > 50 *μ*g/mL) [32]. Apparently, substituting groups R_1_ and R_2_ demonstrated the great role affecting the results.

Two unique bicyclic lactones,** 161-162**, resisted the growth of* M. tuberculosis* with the MIC values of 43.4 *μ*M and 82.1 *μ*M, respectively, but analogs** 163-168** showed no activity. It is possible to note that epoxidation and hydroxylation would not be good circumstances to have positive signals in the activity [38].

### 4.5. Anti-Inflammatory Activity

Inflammation can be seen as a part of the complex biological response of the body tissues to harmful stimuli, such as irradiation, physical damage, metabolic overload, or infection [20]. Nowadays, modern diseases, for instance, cardiovascular and neurodegenerative disorders, are closely related to inflammation. Suppressing NO production is recognized to be a useful strategy for this problem.

On the screening of anti-inflammatory activity, by using mode of lipopolysaccharides (LPS) (1.0 *μ*g/mL)-induced NO production in microglial RAW 264.7 cells, megastigmane glycosides milbasides A-C (**179-181**), alcohols *β*-D-glucopyranoside (*Z*)-3-hexenol (**205**), and (L)-guaiacyl glycerol 2′-*O*-*β*-D-glucopyranoside (**208**), at a concentration of 20.0 *μ*M, together with flavan (−)-epicatechin (**116**) and megastigmane glucoside myrsinionoside D (**184**), at a concentration of 40.0 *μ*M, indicated potent inhibitory activity comparable with or better than positive control sulfuretin (81.3 ± 4.9% at 20.0 *μ*M) [11].

Because of glycosylation, NO inhibitory capacity compound** 208** was generated 2.0-2.5 times higher than those of* erythro-* and* threo*-guaiacylglycerol (**206-207**) (48.4 ± 3.9%) [11].

### 4.6. Antiherpetic Activity

At the dose of 100 *μ*g/mL, methanol extracts from stem and leaf of* M. fragrans* showed the IC_50_ values of 60-80 *μ*g/mL in the antiherpetic experiment against HSV-1 and HSV-2. Meanwhile, in contrast to the inactive results of** 116-118**,** 120-121**,** 124**,** 128-130**,** 135**,** 139**, and** 143-144**, (+)-4-*O*-demethyleusiderin C (**125**) and licarin A (**131**) were found to possess the same IC_50_ values of 62.5-66.7 *μ*g/mL (HSV-1) and 87.5 *μ*g/mL (HSV-2) [9]. It was figured out that 7*S*,8*R*-7.*O*.3′,8.*O*.4′-neolignans should be the best choice for this model. Furthermore, by comparing** 124-125** and** 129-130**, their antiherpetic outcomes closely depended on two functional groups, R_1_ and R_2_ (Figure 4).

### 4.7. Antioxidant Activity

Although phytochemical investigation of* M. wayanadica* species has not yet been performed extensively, its ethanol leaf extract was observed to be equivalent to or better than positive controls in antioxidant assays. For instance, in DPPH assay, the IC_50_ value of 465 *μ*g/mL arising from* M. wayanadica* ethanol leaf extract was comparable with those of standard compounds BHT (570 *μ*g/mL) and BHA (615 *μ*g/mL); with regard to the ferric reducing antioxidant examination, the IC_50_ values for these three objects reached 600 *μ*g/mL, 835 *μ*g/mL, and 870 *μ*g/mL, respectively [50].

### 4.8. Enzyme Acetylcholine Inhibitory Activity

To date, only one research dealt with the use of chemical constituents from* M. thorelii* species exploring the potency of* Miliusa* secondary metabolites in acetylcholinesterase inhibitory activity. The results pointed out that alkaloids (the inhibitory capacity of new oxo-protoberberines** 23-24** and known one** 28** reached the significant range of 27.93%-50.17%) were promising agents rather than flavonoids (the inhibitory percentages of <10%-38.68% were for** 80-83**,** 85-86**,** 88-91**,** 93**,** 95-99**, and** 101-102**) or amides (tested compounds** 201** and** 203** were inactive, <10%) [32].

### 4.9. Cardiac Activity

Chrysosplenol C (**77**), a flavonol, isolated from* M. balansae*, proved to be an essential backbone to induce a positive inotropic effect on rat ventricular myocytes [12]. This compound caused the contractive percentage of ventricular cell and the active percentage of cardiac myosin ATPase up to 53.0 ± 4.07% at 50 *μ*M and 28.1 ± 1.20% at 10 *μ*M, respectively, compared with those of positive control omecamtiv mecarbil [59.3 ± 2.60% at 400 nM and 80.4 ± 2.89% at 10 *μ*M, respectively] [12].

## 5. Conclusion

Taken together,* Miliusa *species have been fully researched in both phytochemical and pharmaceutical aspects, and a general view of the previous results has been outlined in the current paper. This review mostly focused on the knowledge about botanical description, phytochemistry, and biological evaluation. Basic findings might be concluded as below:

- * Miliusa *plants are widely distributed in tropical and subtropical regions, particularly Asia mainland.
- Based on morphological analysis and the heavy support of DNA-barcoding techniques, the number of new* Miliusa *plants discovered increased more often. Up to present, approximately sixty species were identified.
- More than ten* Miliusa* species were highlighted in studying phytochemical and pharmacological aspects. Among them,* Miliusa* plants, collected from Vietnam, Thailand, and China, were major objectives for phytochemical investigations.
- A variety of secondary metabolites have been successfully isolated. In the current paper, we draw a list of twenty-two hundred isolated compounds. Chemical constituents derived from* Miliusa* have fallen into multiple classes of compounds, such as alkaloids, flavonoids, terpenoids, styryls, and lactones, but serial novel derivatives of geranylated homogentisic acid could be seen as biomarkers to recognize* Miliusa *species.
- The geographic factors, environment, and collection time can be responsible for the difference in chemical components of each country. For instance, Thai* M. mollis and M. fragrans *plants established the high amount of lignans and neolignans whereas some Vietnamese* Miliusa* plants were characterized by the rich flavonoids, terpenoids, or miliusanes.
- Naturally occurring isolated compounds and plant extracts of this genus have been subjected to various pharmacological types, but cytotoxic assay seemed to be the main content of previous researches.
- It was also observed that the biological activation or inactivation of tested compounds closely depended on the key role of functional groups in the chemical structures.
- *β*-caryophyllene (10%-20%) was considered as major component in essential oils extracted from Vietnamese and Australian* Miliusa* plants.
- Finally, plant growing proposals, scientific assessments, and extensive phytochemical discoveries on this valuable source ought to be a willingness for drug leads and future pharmaceuticals. Bioactive compounds,* in vitro* and* in vivo* pharmaceutical analyses, clinical applications, and unknown mechanism explanations are expected.

## Figures and Tables

**Table 1:** Chemical constituents from *Miliusa* species.

No	Compounds	Species	References
**Alkaloids**			
**1**	Asimilobine	*M. mollis *twig	[24]
**2**	Coclaurine	*M. balansae* stem	[26]
**3**	Dehydroxylopine	*M. cuneata *stem and leaf	[21]
**4**	1,9-Dihydroxy-2,11-dimethoxy-4,5-dihydro-7-oxoaporphine	*M. cuneata *stem and leaf	[21]
**5**	2,10-Dimethoxy-3,11-dihydroxy-5,6-dihydroprotoberberine	*M. cuneata *stem and leaf	[21]
**6**	*N*,*O*-Dimethylharnovine	*M. cuneata *stem and leaf	[21]
**7**	10-Hydroxyliriodenine	*M.* CF. *banacea *root	[4]
**8**	Isocorydine	*M. velutina *stem bark	[23]
**9**	(+)- Isocorydine *α*-N-oxide	*M. velutina *stem bark	[22]
**10**	Kinabaline	*M. cuneata *stem and leaf	[21]
**11**	Lanuginosine	*M. cuneata *stem and leaf	[21]
**12**	Liriodenine	*M. balansae* stem;* M. cuneata *stem and leaf; * M. mollis *twig, *M. sinensis* leaf and branch; *M. velutina *stem bark	[21, 23–26, 37]
**13**	(+)-Liriotulipiferine	*M. cuneata *stem and leaf	[21]
**14**	10-Methoxyliriodenine (lauterine)	*M.* CF. *banacea *root	[4]
**15**	1-*N*-Methylcoclaurine	*M. balansae* stem	[26]
**16**	*N*-Methylcorydaldine	*M. cuneata *stem and leaf	[21]
**17**	*N*-Methyllindcarpine	*M. cuneata *stem and leaf	[21]
**18**	Miliusacunine A	*M. cuneata* leaf	[7]
**19**	Miliusacunine B	*M. cuneata* leaf	[7]
**20**	Miliusacunine C	*M. cuneata* leaf	[7]
**21**	Miliusacunine D	*M. cuneata* leaf	[7]
**22**	Miliusacunine E	*M. cuneata* leaf	[7]
**23**	Miliusathorine A	*M. thorelii *stem and root	[8]
**24**	Miliusathorine B	*M. thorelii *stem and root	[8]
**25**	Norcorydine	*M. velutina *stem bark	[23]
**26**	(-)-Nordicentrine	*M. cuneata *stem and leaf	[21]
**27**	Norisocorytuberine	*M. cuneata *stem and leaf	[21]
**28**	(−)-Norushinsunine	*M. mollis *twig; * M. thorelii *stem and root	[8, 24]
**29**	Pseudocolumbamine	*M. cuneata *stem and leaf	[21]
**30**	Reticuline	*M. velutina *stem bark	[23]
**31**	Salutarine	*M. cuneata *stem and leaf	[21]
**32**	Wilsonirine	*M. cuneata *stem and leaf	[21]
**Homogentisic acid derivatives**			
**33**	Methyl 2-(1′*β*-geranyl-5′*β*-hydroxy-2′-oxocyclohex-3′-enyl) acetate	*M. umpangensis* leaf; * M. velutina *fruit	[32, 33]
**34**	2-(1′*β*-Geranyl-5′*β*-hydroxy-2′-oxocyclohex-3′-enyl) acetic acid	*M. velutina *fruit and flower	[32]
**35**	Miliusanal	*M. velutina *fruit	[32]
**36**	Miliusanone A	*M. velutina *fruit	[32]
**37**	Miliusanone B	*M. velutina *fruit	[32]
**38**	Miliusanone C	*M. velutina *flower	[32]
**39**	Miliusanone D	*M. velutina *flower	[32]
**40**	(+)-Miliusate	*M. balansae* leaf, branch and stem; *M. sinensis* leaf, twig and flower; *M. umpangensis* leaf	[13, 14, 31, 33, 34]
**41**	(+)-Miliusane I	*M. balansae* stem; * M. sinensis* leaf, twig and flower; * M. umpangensis *leaf	[13, 14, 33]
**42**	(+)-Miliusane II	*M. balansae* stem; * M. sinensis* leaf, twig and flower	[13, 14]
**43**	(+)-Miliusane III	*M. sinensis* leaf, twig and flower	[14]
**44**	(+)-Miliusane IV	*M. sinensis* leaf, twig and flower	[14]
**45**	(+)-Miliusane V	*M. sinensis* leaf, twig and flower	[14]
**46**	(+)-Miliusane VI	*M. sinensis* leaf, twig and flower	[14]
**47**	(+)-Miliusane VII	*M. sinensis* leaf, twig and flower	[14]
**48**	(+)-Miliusane VIII	*M. sinensis* leaf, twig and flower	[14]
**49**	(+)-Miliusane IX	*M. balansae* stem; * M. sinensis* leaf, twig and flower	[13, 14]
**50**	(+)-Miliusane X	*M. sinensis* leaf, twig and flower	[14]
**51**	(+)-Miliusane XI	*M. sinensis* leaf, twig and flower	[14]
**52**	(+)-Miliusane XII	*M. sinensis* leaf, twig and flower	[14]
**53**	(+)-Miliusane XIII	*M. sinensis* leaf, twig and flower	[14]
**54**	(+)-Miliusane XIV	*M. balansae* stem; * M. Sinensis* leaf, twig and flower	[13, 14]
**55**	(+)-Miliusane XV	*M. balansae* stem; * M. Sinensis* leaf, twig and flower	[13, 14]
**56**	(+)-Miliusane XVI	*M. sinensis* leaf, twig and flower	[14]
**57**	(+)-Miliusane XVII	*M. balansae* stem; * M. sinensis* leaf, twig and flower	[13, 14]
**58**	(+)-Miliusane XVIII	*M. sinensis* leaf, twig and flower	[14]
**59**	(+)-Miliusane XIX	*M. sinensis* leaf, twig and flower	[14]
**60**	(+)-Miliusane XX	*M. sinensis* leaf, twig and flower	[14]
**61**	Miliusane XXI	*M. balansae* stem	[13]
**62**	Miliusane XXII	*M. balansae* stem	[13]
**63**	Miliusane XXIII	*M. balansae* stem	[13]
**64**	Miliusane XXIV	*M. balansae* stem	[13]
**65**	Miliusane XXV	*M. balansae* stem	[13]
**66**	Miliusane XXVI	*M. balansae* stem	[13]
**67**	Miliusane XXVII	*M. balansae* stem	[13]
**68**	Miliusane XXVIII	*M. balansae* stem	[13]
**69**	Miliusane XXIX	*M. balansae* stem	[13]
**70**	Miliusane XXX	*M. balansae* stem	[13]
**71**	Miliusane XXXI	*M. balansae* stem	[13]
**72**	(+)-Miliusol	*M. balansae *leaf, stem and branch; * M. cuneata *leaf, *M. sinensis* leaf, twig and flower; *M. umpangensis *leaf	[7, 13, 14, 24, 30]
**73**	Miliusolide^a^	*M. balansae* leaf and branch	[30]
**Flavonoids**			
*Flavones *			
**74**	Artemetin	*M. thorelii *leaf	[8]
**75**	Ayanin (5,3′-dihydroxy-3,7,4′-trimethoxyflavone)	*M. smithiae* leaf and twig;* M. umpangensis* leaf	[33, 36]
**76**	Chrysosplenol B (chrysoplenetin)	*M. balansae* leaf and branch;* M. cuneata* twig	[7, 35]
**77**	Chrysosplenol C	*M. balansae* leaf and branch	[11, 30, 35]
**78**	Chrysosplenol D	*M. umpangensis* leaf	[33]
**79**	3,5-Dihydroxy-7,3′,4′-trimethoxyflavone	*M. sinensis* leaf and branch	[25, 37]
**80**	6,4′-Dihydroxy-3,5,7-trimethoxyflavone	*M. thorelii *stem and root	[8]
**81**	Dimethylmikanin	*M. thorelii *stem and root	[8]
**82**	3,5,6,7,3′,4′-Hexamethoxyflavone	*M. thorelii *leaf	[8]
**83**	5-Hydroxy-3,7-dimethoxy-3′,4′-methylenedioxyflavone	*M. cuneata* leaf and twig; * M. thorelii *stem, root and leaf	[7, 8]
**84**	5-Hydroxy-3,7,4′-trimethoxyflavone	*M. smithiae* leaf and twig	[36]
**85**	5-Hydroxy-3,6,7,4′-tetramethoxyflavone	*M. thorelii *stem and root	[8]
**86**	4′-Hydroxy-3,5,6,7-tetramethoxyflavone	*M. thorelii *stem and root	[8]
**87**	4′-Hydroxy-3,5,7,3′-tetramethoxyflavone	*M. cuneata* leaf	[7]
**88**	Isokanugin	*M. thorelii *leaf	[8]
**89**	3-*O*-Methylkaempferol	*M. thorelii *stem and root	[8]
**90**	Melisimplexin	*M. thorelii *stem, root and leaf	[8]
**91**	Melisimplin	*M. thorelii *leaf	[8]
**92**	Miliufavol	*M. balansae* leaf and branch	[35]
**93**	Miliusathorone	*M. thorelii *stem and root	[8]
**94**	Ombuine	*M. balansae* leaf and branch; * M. umpangensis* leaf	[33, 35]
**95**	Pachypodol	*M. balansae* leaf and branch; *M. cuneata* leaf and twig; * M. thorelii *leaf	[7, 8, 35]
**96**	3,5,7,3′,4′-Pentamethoxyflavone	*M. thorelii *stem, root and leaf	[8]
**97**	Quercetagetin-3,5,7-trimethyl ether	*M. thorelii *stem and root	[8]
**98**	Quercetagetin-3,5,7,3′-tetramethyl ether	*M. thorelii *stem and root	[8]
**99**	Quercetin-3-*O*-methyl ether	*M. thorelii *stem and root	[8]
**100**	Quercetin-3,7-dimethyl ether	*M. thorelii *stem and root;* M. umpangensis* leaf	[8, 33]
**101**	Quercetin-3,5,3′-trimethyl ether	*M. thorelii *stem and root	[8]
**102**	Retusin	*M. thorelii *stem and root	[8]
**103**	Rhamnetin	*M. velutina *leaf	[38]
**104**	Rutin	*M. balansae* leaf; * M. umpangensis* leaf	[11, 33]
**105**	7,3′,4′-Trimethylquercetin	*M. umpangensis* leaf	[33]
*Chalcones*			
**106**	2′,6′-Dihydroxy-4′-Methoxydihydrochalcone	*M. balansae* leaf and branch	[31]
**107**	4′,6′-Dihydroxy-2′,3′,4-trimethoxydihydrochalcone	*M. sinensis* leaf and branch	[37]
**108**	Dihydropashanone	*M. balansae* leaf and branch; * M. sinensis *leaf and branch	[25, 31, 37]
**109**	Pashanone	*M. sinensis* leaf and branch	[6, 25, 37]
*Flavanones*			
**110**	5-Hydroxy-6,7-dimethoxyflavanone (onysilin)	*M. balansae* leaf and branch; *M. sinensis* leaf and branch	[6, 25, 31, 37]
**111**	5-Hydroxy-7,8-dimethoxyflavanone	*M. balansae* leaf and branch; *M. sinensis* leaf and branch	[25, 31, 37]
**112**	5-Hydroxy-7-methoxyflavanone (pinostrobin)	*M. balansae* leaf and branch; *M. sinensis* leaf and branch	[25, 31, 37]
**113**	5-Hydroxy-7,4′-methoxyflavanone	*M. balansae* leaf and branch; *M. sinensis* leaf and branch	[25, 31, 37]
**114**	7-*O*-Methyleriodictyol	*M. velutina *leaf	[38]
**115**	Sakuranetin	*M. velutina *leaf	[38]
*Flavan*			
**116**	(−)-Epicatechin	*M. balansae* leaf; * M. fragrans* leaf and stem;* M. mollis* leaf	[9, 11, 24]
**Lignans and neolignans**			
*Lignans*			
**117**	(+)-3-Hydroxyveraguensin	*M. fragrans *stem	[9]
**118**	(7*S*,8*S*,7′*R*,8′*S*)-3,4,5,3′,4′-Pentamethoxy-7,7′-epoxylignan	*M. fragrans *stem	[9]
**119**	(+)-Syringaresinol	*M. cuneata* leaf	[7]
**120**	Veraguensin	*M. fragrans *stem	[9]
*Neolignans*			
**121**	2-(4-Allyl-2,6-dimethoxyphenoxy)-1-(3,4-dimethoxyphenyl)propane	*M. mollis *leaf	[9]
** 122**	Conocarpan	*M. mollis* twig	[24]
**123**	Decurrenal	*M. mollis *leaf	[10]
**124**	(+)-3-*O*-Demethyleusiderin C [(7*S*,8*R*)-Δ^8′^-3-hydroxy-4,5,5′-trimethoxy-7.*O*.3′,8.*O*.4′-neolignan]	*M. fragrans *leaf	[9]
**125**	(+)-4-*O*-Demethyleusiderin C [(7*S*,8*R*)-Δ^8′^-4-hydroxy-3,5,5′-trimethoxy-7.*O*.3′,8.*O*.4′-neolignan]	*M. fragrans *leaf and stem	[9]
**126**	(2*R*,3*R*)-2,3-Dihydro-2-(4-hydroxy-3-methoxyphenyl)-3-methyl-5-(*E*)-propenylbenzofuran	*M. mollis* twig	[24]
**127**	(2*S*,3*S*)-2,3-Dihydro-2-(4-methoxyphenyl)-3-methyl-5-[1(*E*)-propenyl] benzofuran	*M. mollis* twig	[24]
**128**	(+)-Eusiderin A	*M. fragrans *stem	[9]
**129**	Eusiderin C	*M. fragrans *stem	[9]
**130**	Eusiderin D	*M. fragrans *stem and leaf	[9]
**131**	Licarin A	*M. fragrans *leaf	[9]
**132**	(7*S*,8*S*)-*threo*-Δ^8′^-4-Methoxyneolignan	*M. mollis* twig	[24]
**133**	7-Methoxymiliumollin [(2*R*,3*R*)-5-allyl-2,3-dihydro-2-(4-hydroxyphenyl)-7-methoxy-3-methylbenzofuran]	*M. mollis *leaf	[10]
**134**	3′-Methoxymiliumollin [(2*R*,3*R*)-5-allyl-2,3-dihydro-2-(4-hydroxy-3-methoxyphenyl)-3-methylbenzofuran]	*M. mollis *leaf	[10]
**135**	(−)-4-*O*-Methylmiliusfragrin [(7*R*,8*R*)-Δ^8′^-3,4,5′-trimethoxy-7.*O*.3′,8.*O*.4′-neolignan]	*M. fragrans *stem	[9]
**136**	4′-*O*-Methylmiliumollin [(2*S*,3*S*)-5-allyl-2,3-dihydro-2-(4-methoxyphenyl)-3-methylbenzofuran)]	*M. mollis *leaf	[10]
**137**	(−)-Miliufragranol A [(Δ^7′^-9′-hydroxy-3,4,3′,5′-tetramethoxy-8.*O*.4′-neolignan]	*M. fragrans *stem	[9]
**138**	(−)-Miliufragranol B [(Δ^8′^-4-hydroxy-3,5′-dimethoxy-8.*O*.4′-neolignan]	*M. fragrans *leaf	[9]
**139**	(−)-Miliusfragrin [(7*R*,8*R*)-Δ^8′^-4-hydroxy-3,5′-dimethoxy-7.*O*.3′,8.*O*.4′-neolignan]	*M. fragrans *leaf and stem	[9]
**140**	Miliumollin [(2*R*,3*R*)-5-allyl-2,3-dihydro-2-(4-hydroxyphenyl)-3-methylbenzofuran)]	*M. mollis *leaf	[10]
**141**	Miliumollinone [(2*R*,3*R*)-2,3-dihydro-2-(4-hydroxyphenyl)-3-methyl-5-(2-oxopropyl)-benzofuran]	*M. mollis *leaf	[10]
**142**	Miliusanollin [(7*R*,8*R*)-*threo*-Δ^8′^-7-acetoxy-4-methoxy-8-*O*-4′-neolignan]	*M. mollis *leaf	[10]
**143**	(7S,8R)-7-hydroxy-3,4,3′-trimethoxy-Δ^1,3, 5,1′,3′,5′,8′^-8.*O*.4′-neolignan	*M. fragrans *leaf	[9]
**144**	Virolongin B	*M. fragrans *stem	[9]
**Acetogenins**			
**145**	Acetogenins A	*M. velutina *stem bark	[5]
**146**	Acetogenins B	*M. velutina *stem bark	[5]
**147**	Cananginone A	*M. velutina *stem bark and flower	[32, 40]
**148**	Cananginone B	*M. velutina *stem bark	[40]
**149**	Cananginone C	*M. velutina *stem bark	[40]
**150**	Cananginone D	*M. velutina *stem bark	[40]
**151**	Cananginone E	*M. velutina *stem bark	[40]
**152**	Cananginone F	*M. velutina *stem bark	[40]
**153**	Cananginone G	*M. velutina *stem bark	[40]
**154**	Cananginone H	*M. velutina *stem bark, leaf and flower	[32, 38, 40]
**155**	Cananginone I	*M. velutina *stem bark	[40]
**156**	Miliusolide^a^	*M. velutina *stem bark	[39]
**157**	Miliusolide dihydro derivative	*M. velutina *stem bark	[39]
**158**	Goniothalamusin	*M. velutina *stem bark	[5]
**Lactones**			
**159**	Curcolide	*M. balansae* leaf	[11]
**160**	Serralactone	*M. balansae* leaf	[11]
**161**	Velutinone A	*M. velutina *leaf	[38]
**162**	Velutinone B	*M. velutina *leaf	[38]
**163**	Velutinone C	*M. velutina *leaf	[38]
**164**	Velutinone D	*M. velutina *leaf	[38]
**165**	Velutinone E	*M. velutina *leaf	[38]
**166**	Velutinone F	*M. velutina *leaf	[38]
**167**	Velutinone G	*M. velutina *leaf	[38]
**168**	Velutinone H	*M. velutina *leaf	[38]
**Styryls**			
*Mono-styryl derivatives*			
**169**	3,4-Dimethoxy-6-styryl-pyran-2-one	*M. balansae* leaf and branch	[31]
**170**	(2*E*,5*E*)-2-Methoxy-4-oxo-6-phenyl-hexa-2,5-dienoic acid methyl ester	*M. balansae* leaf and branch	[31]
**171**	Yangonin	*M. velutina *leaf, fruit and flower	[32, 38]
*Bi-styryl derivatives*			
**172**	Miliubisstyryl A	*M. balansae* leaf and branch	[41]
**173**	Miliubisstyryl B	*M. balansae* leaf and branch	[41]
**174**	Velutinindimer A	*M. velutina *leaf, fruit and flower	[32, 38]
**175**	Velutinindimer B	*M. velutina *leaf, fruit and flower	[32, 38]
**176**	Velutinindimer C	*M. velutina *leaf	[38]
**Terpenoids**			
*Norsesquiterpenoids type megastigmanes and megastigmane glycosides *			
**177**	Alangionoside B	*M. balansae *stem	[42]
**178**	Ampelopsisionoside	*M. balansae* leaf	[11]
**179**	Milbaside A	*M. balansae* leaf	[11]
**180**	Milbaside B	*M. balansae* leaf	[11]
**181**	Milbaside C	*M. balansae* leaf	[11]
**182**	Miliusoside C	*M. balansae *stem	[42]
**183**	Myrsinionoside A	*M. balansae* leaf	[11]
**184**	Myrsinionoside D	*M. balansae* leaf	[11]
*Triterpenoid*			
**185**	24-Methylencycloartane-3*β*,21-diol	*M. sinensis* leaf and branch	[25, 37]
**Mono-phenols and mono-phenol glycosides**			
**186**	*bis*(2-Hydroxyphenyl)methyl ether	*M. balansae* leaf and branch	[30]
**187**	Cuchiloside	*M. balansae *stem	[42]
**188**	4-Hydroxybenzonitrile	*M. velutina* fruit	[32]
**189**	4-Hydroxybenzaldehyde	*M. velutina* fruit	[32]
**190**	Icariside D2	*M. mollis* twig	[24]
**191**	Isovanillin	*M. velutina* fruit	[32]
**192**	1-(3-Methylbutyryl)phloroglucinol-glucopyranoside	*M. balansae* leaf	[11]
**193**	Miliusoside A	*M. balansae *stem	[42]
**194**	Miliusoside B	*M. balansae *stem	[42]
**195**	Osmanthuside H	*M. balansae *stem	[42]
**196**	1-(*α*-L-Rhamnosyl-(1→6)-*β*-D-glucopyranosyloxy)-3,4,5-trimethoxybenzene	*M. balansae *stem	[42]
**197**	3,4,5-Trimethoxyphenol-*β*-D-glucopyranoside	*M. balansae *stem	[42]
**198**	Tyrosol-1-*O*-*β*-xylopyranosyl-(1→6)-*O*-*β*-glucopyranoside	*M. mollis* twig	[24]
**Amines and amides**			
**199**	Adenine riboside	*M. balansae* stem	[26]
**200**	Allantoin	*M. balansae* stem	[26]
**201**	*N-trans*-Caffeoyltyramine	*M. cuneata* twig; * M. thorelii *stem and root	[7, 8]
**202**	*N-trans*-Coumaroyltyramine	*M. cuneata* twig	[7]
**203**	*N-trans*-Feruloyltyramine	*M. cuneata* twig; * M. thorelii *stem and root	[7, 8]
**204**	Uridine	*M. balansae* stem	[26]
**Alcohols**			
**205**	*β*-D-Glucopyranoside (*Z*)-3-hexenol	*M. balansae* leaf	[11]
**206**	*erythro*-Guaiacylglycerol	*M. balansae* leaf	[11]
**207**	*threo*-Guaiacylglycerol	*M. balansae* leaf	[11]
**208**	(L)-Guaiacyl glycerol 2′-*O*-*β*-D-glucopyranoside	*M. balansae* leaf	[11]
**209**	*erythro*-1-*C*-Syringylglycerol	*M. balansae* leaf	[11]
**210**	*threo*-1-*C*-Syringylglycerol	*M. balansae* leaf	[11]
**Furfurals**			
**211**	5-Acetyloxymethylfurfural	*M. velutina* fruit	[32]
**212**	5-Hydroxymethylfurfural	*M. velutina* fruit	[32]
**213**	5-Methoxyfurfural	*M. velutina* fruit	[32]
**Others**			
**214**	D-Glucose	*M. balansae* stem	[26]
**215**	Octacosanoic acid	*M. balansae* leaf and branch	[41]
**216**	*β*-Sitosterol	*M. balansae *stem; * M. velutina* fruit	[26, 32]
**217**	*β*-Sitosterol glucoside	*M. balansae *stem;* M. sinensis* leaf and branch	[25, 26]
**218**	Sodium benzoate	*M. balansae* leaf and branch	[30]
**219**	Stigmasterol	*M. sinensis* leaf and branch; * M. velutina* fruit and flower	[25, 32]
**220**	Sucrose	*M. balansae* stem	[26]

^a^The coincidence name

**Table 2:** Essential oils from presentative *Miliusa *species.

Species	Collections	Part Uses	Main constituents	References
*M. baillonii*	Quang Binh-Vietnam	Fresh leaf	Naphthalene (1.0%), bicycloelemene (1.1%), germacrene B (1.2%), germacrene D (1.2%), *δ*-cadinene (1.4%), isolongifolene (1.2%), spathulenol (1.4%), *α*-terpinolene (1.5 %), elemol (1.7%), linalool (2.7%), *β*-elemene (3.5%), *τ*-muurolol (3.8%), *α*-humulene (6.2%), *β*-caryophyllene (10.6%), and *Z*-citral (41.2%)	[28]

*M. sinensis*	Nghean-Vietnam	Dried leaf	(*E*)-*β*-Ocimene (2.4%), aromadendrene (6.6%), *β*-elemene (7.1%), *α*-humulene (7.9%) and *β*-caryophyllene (19.5%)	[29]

*M. brahei*	16°31'S, 145°28'E Queensland-Australia	Leaf	Cubeban-11-ol (1.0%), caryophyllene oxide (1.1%), *α*-copaene (1.2%), *α*-selinene (1.2%), viridiflorene (1.2%), *δ*-cadinene (1.8%), viridiflorol (1.8%), *β*-selinene (2.2%), geraniol (2.3%), (Z)-*β*-ocimene (2.6%), aromadendrene (3.0%), globulol (3.3%),*α*-terpineol (3.5%), spathulenol (3.6%), germacrene D (5.3%), linalool (7.4%), *α*-humulene (11.3%), *β*-caryophyllene (12.8%) and bicyclogermacrene (12.9%)	[27]

*M. horsfieldii*	13°48'S, 143°28'E Queensland-Australia	Leaf	Geraniol (1.0%), globulol (1.2%), cubeban-11-ol (1.3%), bicycloelemene (1.8%), *α*-cadinol (1.9%), allo-aromadendrene (1.9%), viridiflorene (2.5%), bicyclogermacrene (2.5%), *α*-selinene (2.6%), *β*-selinene (2.8%), *δ*-cadinene (3.0%), *α*-humulene (3.4%), linalool (3.8%), *α*-copaene (7.5%), caryophyllene oxide (12.5%) and *β*-caryophyllene (20.2%),	[27]

*M. traceyi*	14°00'S, 143°19'E Queensland-Australia	Leaf	*δ*-Cadinene (1.8%), *α*-humulene (2.4%), spathulenol (2.9%), limonene (3.0%), bicyclogermacrene (3.8%), germacrene D (4.9%), *β*-caryophyllene (13.5%), *β*-pinene (18.6%) and *α*-pinene (18.7%)	[27]

**Table 3:** Cytotoxic results of *Miliusa* components.

No	Inhibitory concentrations (cell lines)	References
*Isolated compound*		

**12**	IC_50_ 2.89 *μ*g/mL (MCF-7), IC_50_ 6.66 *μ*g/mL (LU), IC_50_ 5.23 *μ*g/mL (Hep-G2) and IC_50_ 2.30 *μ*g/mL (KB)	[37]

**18-22**	Inactive (KB and Vero)	[7]

**33**	IC_50_ 26.5 *μ*g/mL (KB), IC_50_ 32.7 *μ*g/mL (MCF-7), IC_50_ 5.8 *μ*g/mL (NCI-H187) and IC_50_ 6.3 *μ*g/mL (Vero)	[32]

**34**	IC_50_ 11.8 *μ*g/mL (KB), IC_50_ > 50.0 *μ*g/mL (MCF-7), IC_50_ 6.1 *μ*g/mL (NCI-H187) and IC_50_ 17.7 *μ*g/mL (Vero)	[32]

**35**	IC_50_ 9.3 *μ*g/mL (KB), IC_50_ 3.6 *μ*g/mL (MCF-7), IC_50_ 40.4 *μ*g/mL (NCI-H187) and IC_50_ 39.1 *μ*g/mL (Vero)	[32]

**36**	IC_50_ 11.9 *μ*g/mL (KB), IC_50_ 23.2 *μ*g/mL (MCF-7), IC_50_ 6.1 *μ*g/mL (NCI-H187) and IC_50_ 16.1 *μ*g/mL (Vero)	[32]

**37**	IC_50_ 17.9 *μ*g/mL (KB), IC_50_ 26.4 *μ*g/mL (MCF-7), IC_50_ 6.2 *μ*g/mL (NCI-H187) and IC_50_ 5.8 *μ*g/mL (Vero)	[32]

**38**	IC_50_ > 50.0 *μ*g/mL (KB, MCF-7, NCI-H187, Vero)	[32]

**39**	IC_50_ > 50.0 *μ*g/mL (KB, MCF-7, NCI-H187, Vero)	[32]

**40**	IC_50_ 1.18 *μ*g/mL (KB), IC_50_ 2.02 *μ*g/mL (Lu1), IC_50_ 1.56 *μ*g/mL (Col2), IC_50_ 3.18 *μ*g/mL (LNCaP), IC_50_ 3.58 *μ*g/mL (MCF-7), IC_50_ 2.89 *μ*g/mL (HUVEC), IC_50_ 0.32 *μ*g/mL (HL60), IC_50_ 2.70 ± 0.09 *μ*M (HCT116), IC_50_ 1.67 ± 0.11 *μ*M (A375) and IC_50_ 6.97 ± 0.16 *μ*M (A549)	[13, 14]

**41**	IC_50_ 1.40 *μ*g/mL (KB), IC_50_ 2.86 *μ*g/mL (Lu1), IC_50_ 2.92 *μ*g/mL (Col2), IC_50_ 5.06 *μ*g/mL (LNCaP), IC_50_ 2.23 *μ*g/mL (MCF-7), IC_50_ 1.79 *μ*g/mL (HUVEC), IC_50_ 0.45 *μ*g/mL (HL60), IC_50_ 3.50 ± 0.02 *μ*M (HCT116), IC_50_ 3.70 ± 0.11 *μ*M (A375) and IC_50_ 4.36 ± 0.40 *μ*M (A549)	[13, 14]

**42**	IC_50_ 5.45 *μ*g/mL (KB), IC_50_ 5.80 *μ*g/mL (Lu1), IC_50_ 9.40 *μ*g/mL (Col2), IC_50_ 19.64 *μ*g/mL (LNCaP), IC_50_ 21.34 *μ*g/mL (MCF-7), IC_50_ 6.55 *μ*g/mL (HUVEC), IC_50_ 1.73 *μ*g/mL (HL60), IC_50_ 17.2 ± 1.86 *μ*M (HCT116), IC_50_ 9.86 ± 0.19 *μ*M (A375) and IC_50_ 18.4 ± 0.35 *μ*M (A549)	[13, 14]

**43**	IC_50_ 1.18 *μ*g/mL (KB), IC_50_ 4.84 *μ*g/mL (Lu1), IC_50_ 4.29 *μ*g/mL (Col2), IC_50_ 5.06 *μ*g/mL (LNCaP), IC_50_ 2.61 *μ*g/mL (MCF-7) and IC_50_ 0.56 *μ*g/mL (HL60)	[14]

**44**	IC_50_ 32.17 *μ*g/mL (KB), IC_50_ 60.43 *μ*g/mL (Lu1), IC_50_ 38.45 *μ*g/mL (Col2), IC_50_ > 62.0 *μ*g/mL (LNCaP), IC_50_ 15.78 *μ*g/mL (MCF-7) and IC_50_ 18.66 *μ*g/mL (HL60)	[14]

**45**	IC_50_ > 55.0 *μ*g/mL (KB, Lu1, Col2, LNCaP and MCF-7) and IC_50_ 52.29 *μ*g/mL (HL60)	[14]

**46**	IC_50_ 3.97 *μ*g/mL (KB), IC_50_ 6.61 *μ*g/mL (Lu1), IC_50_ 4.23 *μ*g/mL (Col2), IC_50_ 5.29 *μ*g/mL (LNCaP) and IC_50_ 4.76 *μ*g/mL (MCF-7)	[14]

**47**	IC_50_ 5.82 *μ*g/mL (KB), IC_50_ 6.16 *μ*g/mL (Lu1), IC_50_ 3.70 *μ*g/mL (Col2), IC_50_ 5.82 *μ*g/mL (LNCaP) and IC_50_ 6.08 *μ*g/mL (MCF-7)	[14]

**48**	IC_50_ 47.35 *μ*g/mL (KB), IC_50_ 63.58 *μ*g/mL (Lu1), IC_50_ 33.44 *μ*g/mL (Col2), IC_50_ 43.38 *μ*g/mL (LNCaP), IC_50_ 26.42 *μ*g/mL (MCF-7) and IC_50_ > 10.9 *μ*g/mL (HUVEC)	[14]

**49**	IC_50_ > 57.4 *μ*g/mL (KB, Lu1, LNCaP), IC_50_ 46.01 *μ*g/mL (Col2), IC_50_ 52.56 *μ*g/mL (MCF-7), IC_50_ 13.3 ± 0.62 *μ*M (HCT116), IC_50_ 7.24 ± 0.81 *μ*M (A375) and IC_50_ 18.3 ± 2.54 *μ*M (A549)	[13, 14]

**50/51**	IC_50_ 5.22 *μ*g/mL (KB), IC_50_ 21.44 *μ*g/mL (Lu1), IC_50_ 8.03 *μ*g/mL (Col2), IC_50_ 29.56 *μ*g/mL (LNCaP), IC_50_ 5.03 *μ*g/mL (MCF-7) and IC_50_ 3.28 *μ*g/mL (HL60)	[14]

**52/53**	IC_50_ 54.97 *μ*g/mL (KB), IC_50_ 9.31 *μ*g/mL (Lu1), IC_50_ 13.43 *μ*g/mL (Col2), IC_50_ 51.82 *μ*g/mL (LNCaP) and IC_50_ 12.18 *μ*g/mL (MCF-7)	[14]

**54/55**	IC_50_ 5.28 *μ*g/mL (KB), IC_50_ 7.46 *μ*g/mL (Lu1), IC_50_ 5.36 *μ*g/mL (Col2), IC_50_ 27.62 *μ*g/mL (LNCaP), IC_50_ 10.06 *μ*g/mL (MCF-7), IC_50_ 3.30 ± 0.06 *μ*M (HCT116), IC_50_ 3.38 ± 0.09 *μ*M (A375) and IC_50_ 10.4 ± 0.32 *μ*M (A549)	[13, 14]

**56**	IC_50_ 6.11 *μ*g/mL (KB), IC_50_ 19.94 *μ*g/mL (Lu1), IC_50_ 3.89 *μ*g/mL (Col2), IC_50_ 6.11 *μ*g/mL (LNCaP) and IC_50_ 6.39 *μ*g/mL (MCF-7)	[14]

**57**	IC_50_ 6.71 *μ*g/mL (KB), IC_50_ 14.94 *μ*g/mL (Lu1), IC_50_ 9.48 *μ*g/mL (Col2), IC_50_ 23.95 *μ*g/mL (LNCaP), IC_50_ 10.99 *μ*g/mL (MCF-7), IC_50_ 4.20 ± 0.30 *μ*M (HCT116), IC_50_ 4.25 ± 0.05 *μ*M (A375) and IC_50_ 20.8 ± 1.24 *μ*M (A549)	[13, 14]

**58**	IC_50_ 3.07 *μ*g/mL (KB), IC_50_ 1.82 *μ*g/mL (Lu1), IC_50_ 2.26 *μ*g/mL (Col2), IC_50_ 2.41 *μ*g/mL (LNCaP), IC_50_ 3.01 *μ*g/mL (MCF-7) and IC_50_ 0.63 *μ*g/mL (HL60)	[14]

**59**	IC_50_ 2.61 *μ*g/mL (KB), IC_50_ 1.82 *μ*g/mL (Lu1), IC_50_ 2.01 *μ*g/mL (Col2), IC_50_ 1.73 *μ*g/mL (LNCaP) and IC_50_ 2.26 *μ*g/mL (MCF-7)	[14]

**60**	IC_50_ > 59.0 *μ*g/mL (KB, Lu1, Col2, LNCaP and MCF-7) and IC_50_ 57.01 *μ*g/mL (HL60)	[14]

**61**	IC_50_ > 65.0 *μ*g/mL (HCT116, A375, A549)	[13]

**62/63**	IC_50_ 9.80 ± 0.51 *μ*M (HCT116), IC_50_ 6.96 ± 0.23 *μ*M (A375) and IC_50_ 24.3 ± 1.42 *μ*M (A549)	[13]

**64/65**	IC_50_ > 52.9 *μ*M (HCT116, A375 and A549)	[13]

**66/67**	IC_50_ > 55.0 *μ*M (HCT116, A375 and A549)	[13]

**68**	IC_50_ 4.10 ± 0.13 *μ*M (HCT116), IC_50_ 3.60 ± 0.33 *μ*M (A375) and IC_50_ 7.15 ± 0.18 *μ*M (A549)	[13]

**69**	IC_50_ > 57.5 *μ*M (HCT116, A375 and A549)	[13]

**70**	IC_50_ 13.0 ± 0.17 *μ*M (HCT116), IC_50_ 10.4 ± 0.66 *μ*M (A375) and IC_50_ 24.5 ± 1.56 *μ*M (A549)	[13]

**71**	IC_50_ 18.3 ± 0.59 *μ*M (HCT116), IC_50_ 11.6 ± 1.45 *μ*M (A375) and IC_50_ 18.0 ± 2.80 *μ*M (A549)	[13]

**72**	IC_50_ 10.2 ± 0.1 *μ*M (KB)^b^, IC_50_ 13.5 ± 0.5 *μ*M (Vero), IC_50_ 2.00 ± 0.16 *μ*M (HCT116), IC_50_ 1.50 ± 0.15 *μ*M (A375), IC_50_ 2.45 ± 0.24 *μ*M (A549), IC_50_ 1.18 *μ*g/mL (KB)^c^, IC_50_ 1.64 *μ*g/mL (Lu1), IC_50_ 1.35 *μ*g/mL (Col2), IC_50_ 1.78 *μ*g/mL (LNCaP), IC_50_ 3.09 *μ*g/mL (MCF-7), IC_50_ 1.32 *μ*g/mL (HUVEC) and IC_50_ 0.66 *μ*g/mL (HL60)	[7]^b^; [13], [14]^c^

**75**	ED_50_ 3.6 *μ*g/mL (P-388), ED_50_ 0.76 *μ*g/mL (Col2), ED_50_ 0.68 *μ*g/mL (MCF-7) ED_50_ 16.08 *μ*g/mL (ASK), ED_50_ 2.81 *μ*g/mL (Hek293) and Inactive (KB, Lu-1 and T24)	[36]

**76**	IC_50_ 4.6 *μ*g/mL (KB), IC_50_ 0.93 *μ*g/mL (Hep-G2) and IC_50_ > 5.0 *μ*g/mL (RD)	[35]

**77**	IC_50_ 4.3 *μ*g/mL (KB), IC_50_ 0.57 *μ*g/mL (Hep-G2) and IC_50_ 2.09 *μ*g/mL (RD)	[35]

**79** and **107**	IC_50_ > 128.0 *μ*g/mL (MCF-7, LU, Hep-G2 and KB)	[37]

**83** and **87**	Inactive (KB and Vero)	[7]

**94**	> 5.0 *μ*g/mL (KB and RD) and 1.5 *μ*g/mL (Hep-G2)	[35]

**95**	IC_50_ 0.7 *μ*g/mL (KB), IC_50_ 0.55 *μ*g/mL (Hep-G2) and IC_50_ 3.01 *μ*g/mL (RD)	[35]

**116**	Inactive (KB, MCF-7 and NCI-H187)	[9]

**119**	Inactive (KB and Vero)	[7]

**123**	IC_50_ 137.4 *μ*M (KB), IC_50_ 169.1 *μ*M (MCF-7) and IC_50_ 94.7 *μ*M (NCI-H178)	[10]

**124**	IC_50_ 20.0 *μ*g/mL (KB), IC_50_ 21.0 *μ*g/mL (MCF-7) and IC_50_ 17.1 *μ*g/mL (NCI-H178)	[9]

**125**	IC_50_ 17.9 *μ*g/mL (KB), IC_50_ 28.4 *μ*g/mL (MCF-7) and IC_50_ 15.9 *μ*g/mL (NCI-H178)	[9]

**130**	IC_50_ 18.4 *μ*g/mL (KB), IC_50_ 22.6 *μ*g/mL (MCF-7) and IC_50_ 20.6 *μ*g/mL (NCI-H178)	[9]

**131**	IC_50_ 12.9 *μ*g/mL (KB), IC_50_ 45.6 *μ*g/mL (MCF-7) and IC_50_ 16.7 *μ*g/mL (NCI-H178)	[9]

**134**	IC_50_ 31.4 *μ*M (KB), IC_50_ 56.2 *μ*M (MCF-7) and IC_50_ 61.3 *μ*M (NCI-H178)	[10]

**139**	IC_50_ 23.8 *μ*g/mL (KB), IC_50_ 24.4 *μ*g/mL (MCF-7) and IC_50_ 16.7 *μ*g/mL (NCI-H178)	[9]

**140**	IC_50_ 27.2 *μ*M (KB), IC_50_ 71.9 *μ*M (MCF-7) and IC_50_ 95.3 *μ*M (NCI-H178)	[10]

**141**	IC_50_ 95.9 *μ*M (KB), IC_50_ 142.7 *μ*M (MCF-7) and IC_50_ 115.9 *μ*M (NCI-H178)	[10]

**143**	IC_50_ 14.4 *μ*g/mL (KB), IC_50_ 13.0 *μ*g/mL (MCF-7) and IC_50_ 12.7 *μ*g/mL (NCI-H178)	[9]

**145**	LD_90_ 7.1 *μ*g/mL	[5]

**146**	LD_90_ 14.1 *μ*g/mL	[5]

**147**	IC_50_ 99.0 *μ*M (KB), Inactive (MCF7) and IC_50_ 48.9 *μ*M (NCI-H187)	[40]

**148**	IC_50_ 67.4 *μ*M (KB), IC_50_ 93.7 *μ*M (MCF-7) and IC_50_ 60.7 *μ*M (NCI-H187)	[40]

**149**	IC_50_ 57.2 *μ*M (KB), IC_50_ 84.8 *μ*M (MCF-7) and IC_50_ 66.3 *μ*M (NCI-H187)	[40]

**150**	IC_50_ 79.8 *μ*M (KB), IC_50_ 126.3 *μ*M (MCF7) and IC_50_ 61.1 *μ*M (NCI-H187)	[40]

**151**	IC_50_ 45.2 *μ*M (KB), IC_50_ 16.6 *μ*M (MCF-7) and IC_50_ 70.2 *μ*M (NCI-H187)	[40]

**152**	IC_50_ 33.9 *μ*M (KB), IC_50_ 67.3 *μ*M (MCF7) and IC_50_ 27.0 *μ*M (NCI-H187)	[40]

**153**	IC_50_ 112.6 *μ*M (KB), Inactive (MCF-7) and IC_50_ 66.7 *μ*M (NCI-H187)	[40]

**154**	IC_50_ 59.9 *μ*M (KB), IC_50_ 92.0 *μ*M (MCF7) and IC_50_ 28.6 *μ*M (NCI-H187)	[40]

**155**	IC_50_ 43.3 *μ*M (KB), IC_50_ 129.7 *μ*M (MCF-7) and IC_50_ 32.3 *μ*M (NCI-H187)	[40]

**158**	LD_90_ 20.0 *μ*g/mL	[5]

**161**	IC_50_ 4.0 *μ*M (KB), IC_50_ 4.8 *μ*M (MCF-7), IC_50_ 4.2 *μ*M (NCI-H187) and IC_50_ 5.8 *μ*M (Vero)	[38]

**162**	IC_50_ 9.6 *μ*M (KB), IC_50_ 12.9 *μ*M (MCF-7), IC_50_ 6.5 *μ*M (NCI-H187) and IC_50_ 8.8 *μ*M (Vero)	[38]

**163**	IC_50_ 12.9 *μ*M (KB), IC_50_ 10.9 *μ*M (MCF-7), IC_50_ 11.4 *μ*M (NCI-H187) and IC_50_ 10.3 *μ*M (Vero)	[38]

**164**	IC_50_ 10.5 *μ*M (KB), IC_50_ 15.2 *μ*M (MCF-7), IC_50_ 8.7 *μ*M (NCI-H187) and IC_50_ 11.7 *μ*M (Vero)	[38]

**166**	IC_50_ 14.5 *μ*M (KB), IC_50_ 20.7 *μ*M (MCF-7), IC_50_ 11.5 *μ*M (NCI-H187) and IC_50_ 11.2 *μ*M (Vero)	[38]

**167**	IC_50_ 24.1 *μ*M (KB), IC_50_ 21.0 *μ*M (MCF-7), IC_50_ 14.7 *μ*M (NCI-H187) and IC_50_ 17.9 *μ*M (Vero)	[38]

**168**	IC_50_10.5 *μ*M (KB), IC_50_ 11.9 *μ*M (MCF-7), IC_50_ 6.8 *μ*M (NCI-H187) and IC_50_ 18.2 *μ*M (Vero)	[38]

**174-176**	Inactive (KB, MCF-7, NCI-H187 and Vero)	[38]

**201-203**	Inactive (KB and Vero)	[7]

*Plant extracts*		

*n*-Hexane extract of *M. sinensis*	IC_50_ 86.6 *μ*g/mL (MCF-7), IC_50_ 78.33 *μ*g/mL (LU), IC_50_ 36.72 *μ*g/mL (Hep-G2) and IC_50_ 82.04 *μ*g/mL (KB)	[37]

Ethyl acetate extract of *M. sinensis*	IC_50_ 72.52 *μ*g/mL (MCF-7), IC_50_ 75.09 *μ*g/mL (LU), IC_50_ 42.50 *μ*g/mL (Hep-G2) And IC_50_ 59.13 *μ*g/mL (KB)	[37]

*n*-Butanol extract of *M. sinensis*	IC_50_ > 128.0 *μ*g/mL (MCF-7 and KB)	[37]

*n*-Hexane extract of *M. smithiae*	ED_50_ 9.07 *μ*g/mL (P-388), ED_50_ 12.0 *μ*g/mL (KB), ED_50_ 8.53 *μ*g/mL (Col2), ED_50_ 1.16 *μ*g/mL (MCF-7), ED_50_ 11.98 *μ*g/mL (Lu1), ED_50_ 13.31 *μ*g/mL (T24), ED_50_ 11.6 *μ*g/mL (ASK) and ED_50_ 6.74 *μ*g/mL (Hek293)	[36]

Ethyl acetate extract of *M. smithiae*	ED_50_ 2.07 *μ*g/mL (P-388), ED_50_ 5.45 *μ*g/mL (KB), ED_50_ 1.98 *μ*g/mL (Col2), ED_50_ 0.3 *μ*g/mL (MCF-7), ED_50_ 5.85 *μ*g/mL (Lu1), ED_50_ 3.29 *μ*g/mL (T24), ED_50_ 3.83 *μ*g/mL (ASK) and ED_50_ < 4.0 *μ*g/mL (Hek293)	[36]

Methanol and acetone extracts of *M. smithiae*	Inactive (P-388, KB, Col2, MCF-7, Lu1, T24, ASK and Hek293)	[36]

^b, c^The results derived from different models.

## Abbreviations

HPLC: High performance liquid chromatography
GC-MS: Gas chromatography-mass spectrum
NMR: Nuclear magnetic resonance
UV-Vis: Ultraviolet-visible
IR: Infrared
OR: Optical rotation
CD: Circular dichroism
LPS: Lipopolysaccharide
DPPH: 1,1-Diphenyl-2-picrylhydrazyl
BHA: Butylated hydroxyanisole
BHT: Butylated hydroxytoluene
HSV-1 and HSV-2: Herpes simplex virus-1 and herpes simplex virus-2
MCF-7: Human breast carcinoma cell line
A549 and LU: Human lung carcinoma cell lines
Hep-G2, KB: Human oral epidermoid carcinoma cell line
Vero: Kidney epithelial cell line
NCI-H187, Col2, and HCT116: Human colon carcinoma cell lines
LNCaP: Human prostate carcinoma cell line
HUVEC: Human umbilical vein endothelial cell line
HL60: Human promyelocytic leukemia cell line
A375: Human melanoma carcinoma cell line
P-388: Human murine lymphocytic leukemia cell line
ASK: Rat glioma cell line
Hek293: Noncancerous human embryonic kidney cell line
T24: Human urinary bladder cancer cell line.
